# Population genetics of transposable element load: A mechanistic account of observed overdispersion

**DOI:** 10.1371/journal.pone.0270839

**Published:** 2022-07-14

**Authors:** Ronald D. Smith, Joshua R. Puzey, Gregory D. Conradi Smith

**Affiliations:** 1 Department of Applied Science, William & Mary, Williamsburg, VA, United States of America; 2 Department of Biology, William & Mary, Williamsburg, VA, United States of America; University of Bari: Universita degli Studi di Bari Aldo Moro, ITALY

## Abstract

In an empirical analysis of transposable element (TE) abundance within natural populations of *Mimulus guttatus* and *Drosophila melanogaster*, we found a surprisingly high variance of TE count (e.g., variance-to-mean ratio on the order of 10 to 300). To obtain insight regarding the evolutionary genetic mechanisms that underlie the overdispersed population distributions of TE abundance, we developed a mathematical model of TE population genetics that includes the dynamics of element proliferation and purifying selection on TE load. The modeling approach begins with a master equation for a birth-death process and extends the predictions of the classical theory of TE dynamics in several ways. In particular, moment-based analyses of population distributions of TE load reveal that overdispersion is likely to arise via copy-and-paste proliferation dynamics, especially when the elementary processes of proliferation and excision are approximately balanced. Parameter studies and analytic work confirm this result and further suggest that overdispersed population distributions of TE abundance are probably not a consequence of purifying selection on total element load.

## Introduction

The genomics revolution has revealed that a significant portion of eukaryotic genomes consists of transposable elements (TEs, also called mobile DNA elements). Notable examples include the human and maize genomes, 44% and 85% of which are TE sequences [1, 2]. Various mobility mechanisms enable TEs to proliferate and/or change position within a genome (transposition). The effect of TEs can range from having little to no consequence on phenotype to being powerful mutagens [3]. In addition to the innate tendency of TEs to proliferate, the distinction between autonomous and non-autonomous TEs, mutations leading to nonactive elements, and factors such as recombination, epigenetics, and selection contribute to their complex genomic distribution and demography [4, 5]. The population structure of TE families may represent an evolutionary equilibrium between proliferation and selection, i.e., transposition-selection balance. Alternatively, increased proliferation rates (transposition bursts) may on occasion generate TE families that have not had enough time to reach evolutionary equilibrium [6–8]. While it is clear that TEs have been an integral part of the long-term evolution of genome architecture, much about the role of TEs in evolution remains unknown. Knowledge of the dynamics of TE abundance in natural populations is an important step toward an increasing understanding of how genomes evolve.

The classical population genetic theory of TEs used a combination of mathematical analysis, computer simulation, and a limited amount of experimental data, to give theoretical insight into TE dynamics and demographics [9]. This modeling considered a single family of TEs with a drift-diffusion representation of TE proliferation, with either no selection or weak selection acting on total TE copy number. This modeling approach has informed our understanding of the population genetics of TEs for several decades. However, the classical theory does not reproduce experimentally observed within-population variances that often greatly exceed the population mean. The cause of this discrepancy is that the classical approach assumes a binomial distribution of within-population TE loads, which constrains the population variance to be no greater than the population mean.

This paper begins with a brief review of classical TE population genetics. This is followed by an analysis of TE demography derived from genome-sequence data of two natural populations (*Mimulus guttatus* and *Drosophila melanogaster*). Notably, in both cases, we observe that the within-population variance of TE load is highly overdispersed. Because these empirical results violate the predictions of classical TE modeling, we developed a master equation formulation of the population distribution of TE loads in a large randomly mating population. This alternative population genetic framework simultaneously and self-consistently predicts both the mean and variance of within-population TE load. This model of TE population genetics is then interrogated to identify evolutionary genetic mechanisms that influence the population variance of TE load. Moment-based analyses of time-dependent and equilibrium population distributions of TE load reveal that overdispersion may arise via copy-and-paste proliferation dynamics, especially when the elementary processes of proliferation and excision are first-order and balanced. Parameter studies and analytic work confirm this result and further suggest that overdispersed population distributions of TE abundance are probably not a consequence of purifying selection on total element load.

### Classical population genetics of TEs

In classical TE population genetics a chromosome is modeled as a finite set of *m* available insertion sites (loci) per haploid genome, each of which can either be occupied by a transposable element (or not) [9–13]. For a single family of TEs, the state of an infinite diploid population at a given chromosomal site *i*, for *i* = 1, 2, …, *m*, is described by its frequency, *x*_*i*_, where 0 ≤ *x*_*i*_ ≤ 1. Assuming insertion sites exhibit no linkage disequilibrium, the set of frequencies, ${\left\{x_{i}\right\}}_{i=1}^{m}$, describes the state of the population. The mean copy number of TEs per individual is $\bar{n}=2\sum_{i=1}^{m}x_{i}$, where the factor of 2 accounts for diploidy.

The evolutionarily neutral version of the classical theory includes two processes affecting TE load (gain and loss). Gain of TEs is represented by a proliferation rate (per individual per element per generation) in the germ line of an individual with *n* elements. This proliferation rate, denoted *u*_*n*_, is typically assumed to be a decreasing function of TE load (*du*_*n*_/*dn* < 0). Loss of TEs is represented by a first-order excision rate constant (per individual per element per generation) denoted by *ν*. The change (per generation) in the mean TE copy number per individual is thus

$$\begin{array}{c} \Delta \bar{n}=E\left[nu_{n}\right]-\nu \bar{n}\;, \end{array}$$ (1)

where **n** is the diploid TE load of a randomly sampled individual, the expected value is taken over individuals in the population, and $\bar{n}=E\left[n\right]$ is the population mean of TE copy number. Expanding Eq (1) around the mean TE load gives the following second-order approximation,

$$\begin{array}{c} \Delta \bar{n}\approx \bar{n}\left(u_{\bar{n}}-\nu \right)+\frac{V_{\bar{n}}}{2}(2\frac{du_{\bar{n}}}{d\bar{n}}+\bar{n}\frac{d^{2}u_{\bar{n}}}{d{\bar{n}}^{2}})\;, \end{array}$$ (2)

where $V_{\bar{n}}$ denotes the population variance in TE copy number. If the higher order terms that scale the population variance are negligible, the change in mean TE copy number per generation is $\Delta \bar{n}\approx \bar{n}\left(u_{\bar{n}}-\nu \right)$. For this neutral model of TE population dynamics, one concludes that $\bar{n}$ will approach a (stable) equilibrium value satisfying $u_{\bar{n}}\approx \nu$ provided $du_{\bar{n}}/d\bar{n}<0$.

To extend this model of TE population genetics to include the effect of natural selection, it is customary to assume a viability function, *w*_*n*_, that is a decreasing function of genome-wide TE load (*dw*_*n*_/*dn* < 0). Approximating the mean fitness of the population ($E[w_{n}]$) by the fitness of an individual with an average number of copies ($w_{\bar{n}}$), Eq (2) can be extended to include the effect of selection on TE load [13],

$$\begin{array}{c} \Delta \bar{n}\approx V_{\bar{n}}\;\frac{d\;\text{ln}w_{\bar{n}}}{d\bar{n}}+\bar{n}\left(u_{\bar{n}}-\nu \right)+\frac{V_{\bar{n}}}{2}(2\frac{du_{\bar{n}}}{d\bar{n}}+\bar{n}\frac{d^{2}u_{\bar{n}}}{d{\bar{n}}^{2}})\;. \end{array}$$ (3)

As a specific example, consider the proliferation rate function *u*_*n*_ = *ξ*_0_/*n* with *ξ*_0_ > 0 and the selection function *w*_*n*_ = *e*^−*γn*^ for *γ* > 0 (viability is a decreasing function of TE copy number). Because *du*_*n*_/*dn* = −*ξ*_0_/*n*^2^ and *d*^2^ *u*_*n*_/*dn*^2^ = 2*ξ*_0_/*n*^3^, the higher order terms involving derivatives of *u*_*n*_ evaluate to zero. Consequently, Eq (3) becomes

$$\begin{array}{c} \Delta \bar{n}\approx V_{\bar{n}}\;\frac{d\;\text{ln}\;w_{\bar{n}}}{d\bar{n}}+\xi_{0}-\nu \bar{n}\;. \end{array}$$

Substituting $d\;\text{ln}\;w_{\bar{n}}/d\bar{n}=-\gamma$ and setting $\Delta \bar{n}=0$ gives $0=-\gamma V_{\bar{n}}+\xi_{0}-\nu \bar{n}$. Solving for the equilibrium mean TE load gives,

$$\begin{array}{c} \bar{n}=\frac{\xi_{0}-\gamma V_{\bar{n}}}{\nu }\;. \end{array}$$ (4)

This result is biologically meaningful for $\xi_{0}>\gamma V_{\bar{n}}$. As expected, the equilibrium TE load is an increasing function of the proliferation rate constant, *ξ*_0_, and a decreasing function of the excision rate constant, *ν*. Furthermore, stronger selection against TE load (greater *γ*) decreases the mean value of the equilibrium TE load in the population.

### Population variance in the classical model

Analysis of the classical model of TE population genetics proceeds in an *ad hoc* manner by making further assumptions regarding the population variance, $V_{\bar{n}}$, which is a parameter in Eqs (2)–(4). For example, one may assume [9] the population variance takes the form

$$\begin{array}{c} V_{\bar{n}}=\bar{n}(1-\frac{\bar{n}}{2m})-2m\sigma_{x}^{2}+4\sum_{i<j}D_{ij}\;, \end{array}$$ (5)

where *D* is a matrix of linkage disequilibrium coefficients [14], and $\sigma_{x}^{2}=\frac{1}{m}\sum_{i=1}^{m}{\left(x_{i}-\bar{x}\right)}^{2}$ is the variance in element frequency across loci (see Sec 1 in S1 Text). If one further assumes that linkage effects are small enough to be ignored, then

$$\begin{array}{c} V_{\bar{n}}\approx \bar{n}(1-\frac{\bar{n}}{2m})-2m\sigma_{x}^{2}\;. \end{array}$$ (6)

For a large enough population, one expects the variance in element frequency across loci to be eventually become negligible, $\sigma_{x}^{2}\to 0$ and, consequently, the equilibrium population variance of TE load should approach that of a binomial distribution,

$$\begin{array}{c} V_{\bar{n}}\approx \bar{n}(1-\frac{\bar{n}}{2m})\;. \end{array}$$ (7)

In that case, assuming occupiable loci are not limiting ($\bar{n}<<2m$), the population variance is well-approximated by the mean ($V_{\bar{n}}\approx \bar{n}$). Substituting this value into Eq (4), the classical model indicates that the equilibrium TE load will be

$$\begin{array}{c} \bar{n}=\frac{\xi_{0}}{\gamma +\nu }\;. \end{array}$$ (8)

As in Eq (4), the equilibrium TE load is an increasing function of the proliferation rate constant (*ξ*_0_), and a decreasing function of both the excision rate constant (*ν*) and the strength of selection against TE load (*γ*).

The classical model, Eqs (3)–(8), has informed expectations regarding the population genetics of TEs for several decades. For example, an extension of this classical theory predicts that in a finite population of effective size *N*_*e*_, the the stationary distribution of TE frequency (*x*) will take the form *ρ*(*x*) ∝ *x*^*a*−1^(1 − *x*)^*b*−1^ where $a=4N_{e}\bar{n}u_{\bar{n}}/\left(2m-\bar{n}\right)$ and $b=4N_{e}(\nu +|d\;\text{ln}\;w_{\bar{n}}/d\bar{n}\left|\right)$ [11]. For *u*_*n*_ = *ξ*_0_/*n*, *w*_*n*_ = *e*^−*γn*^, and $\bar{n}<<2m$, this gives *a* = 4*N*_*e*_*ξ*_0_ and *b* = 4*N*_*e*_(*ν* + *γ*). On the other hand, the classical approach to modeling TE population genetics has obvious limitations. For one thing, the derivation and analysis of the classical model makes assumptions about the population variance, $V_{\bar{n}}$ in Eqs (5)–(7), that may not be consistent with experimental observations (see Results). Furthermore, the population variance of TE load ought to be an emergent property of a model constructed for the purpose of understanding the population genetics of TEs, rather than a modeling assumption that is imposed upon a preexisting framework, as in Eq (7).

The remainder of this paper addresses these two issues in detail. We begin with empirical evidence that population variance of TEs is neither binomial nor well-approximated by the mean. This motivates the presentation of an alternative population genetic framework that, simultaneously and self-consistently, predicts both the population variance and the mean TE load. This model of TE population genetics is then interrogated to identify evolutionary genetic mechanisms that influence the population variance of TE load.

## Results

### Dispersion of TE loads in the classical model

In the classical modeling of TE population genetics discussed above, analytical results are obtained by assuming a randomly mating population with a binomial distribution of TE loads,

$$\begin{array}{c} n∼\text{Binomial}(2m,\bar{n}/2m)\;, \end{array}$$ (9)

with mean $E\left[n\right]=\bar{n}$ and variance $\text{Var}\left[n\right]=\bar{n}\left(1-\bar{n}/2m\right)$. A simple measure of the variability of TE load within a population is the *index of dispersion* (Fano factor) given by

$$\begin{array}{c} \text{Fano}\left[n\right]=\frac{\text{Var}[n]}{E[n]}\;. \end{array}$$ (10)

Substituting the mean and variance of the binomial distribution into Eq (10), it is apparent that the classical model of TE population genetics predicts (i.e., assumes) a Fano factor that is less than one,

$$\begin{array}{c} \text{Fano}\left[n\right]=1-\frac{\bar{n}}{2m}<1\;. \end{array}$$

In fact, when the number of sites occupied by TEs is small compared to the total number of occupiable loci (*m* → ∞ with $\bar{n}$ fixed), the Fano factor approaches one from below (Fano[n]→1). In this limit, the binomial distribution of Eq (9) is well-approximated by $n∼\text{Poisson}(\bar{n})$. If it were the case that the TE load within a population were Poisson distributed, then the mean and variance of TE load would be equal ($E\left[n\right]=\text{Var}\left[n\right]=\bar{n}$) and the index of dispersion would be Fano[n]=1. With our expectations set by this prediction of classical modeling, empirical observations of a Fano factor greater than one (Fano[n]>1) would indicate *overdispersion* of TE load within a population.

### Overdispersion of empirical TE counts

**Fig 1:**
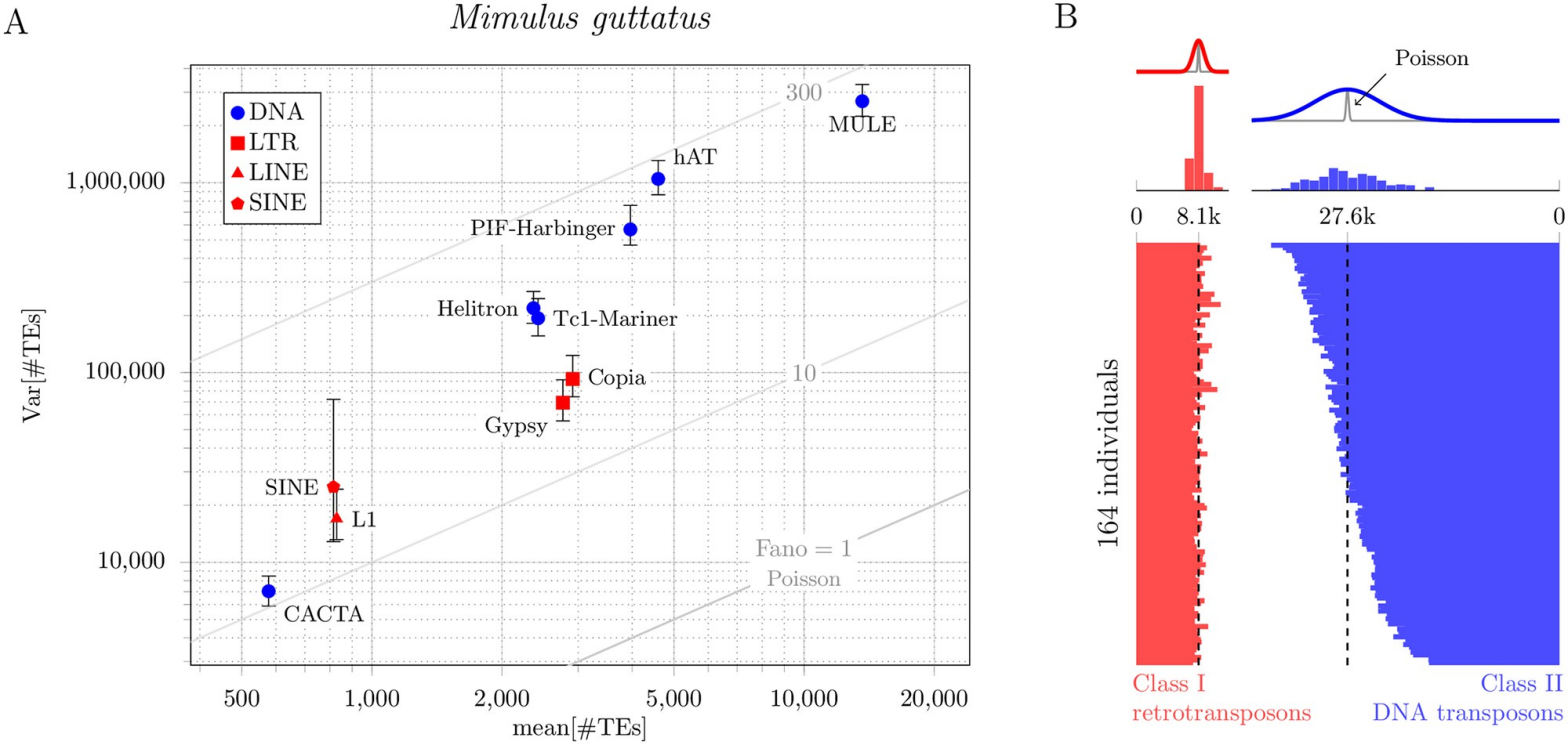
Empirically observed distributions of TE copy number in a *Mimulus guttatus* population. A: Mean-variance plot of TE copy number for 164 individuals compared to theoretical expectation (Fano = 1, Poisson diagonal line). For each of ten different families of TEs, the index of dispersion is in the range 10<Fano[n]<300. For each family, the vertical bars show 95% bootstrap confidence interval of the population variance of TE load. B: TE counts separated by class (red, Class I, retrotransposon; blue, Class II, DNA transposon). The variability in TE load can be observed in the counts from individuals (bottom) as well as histograms (top). Overdispersion is apparent in the deviation of the observed counts (red and blue histograms) from the corresponding Poisson distributions (gray lines). The vertical dashed lines show the population mean of TE load.

**Fig 2:**
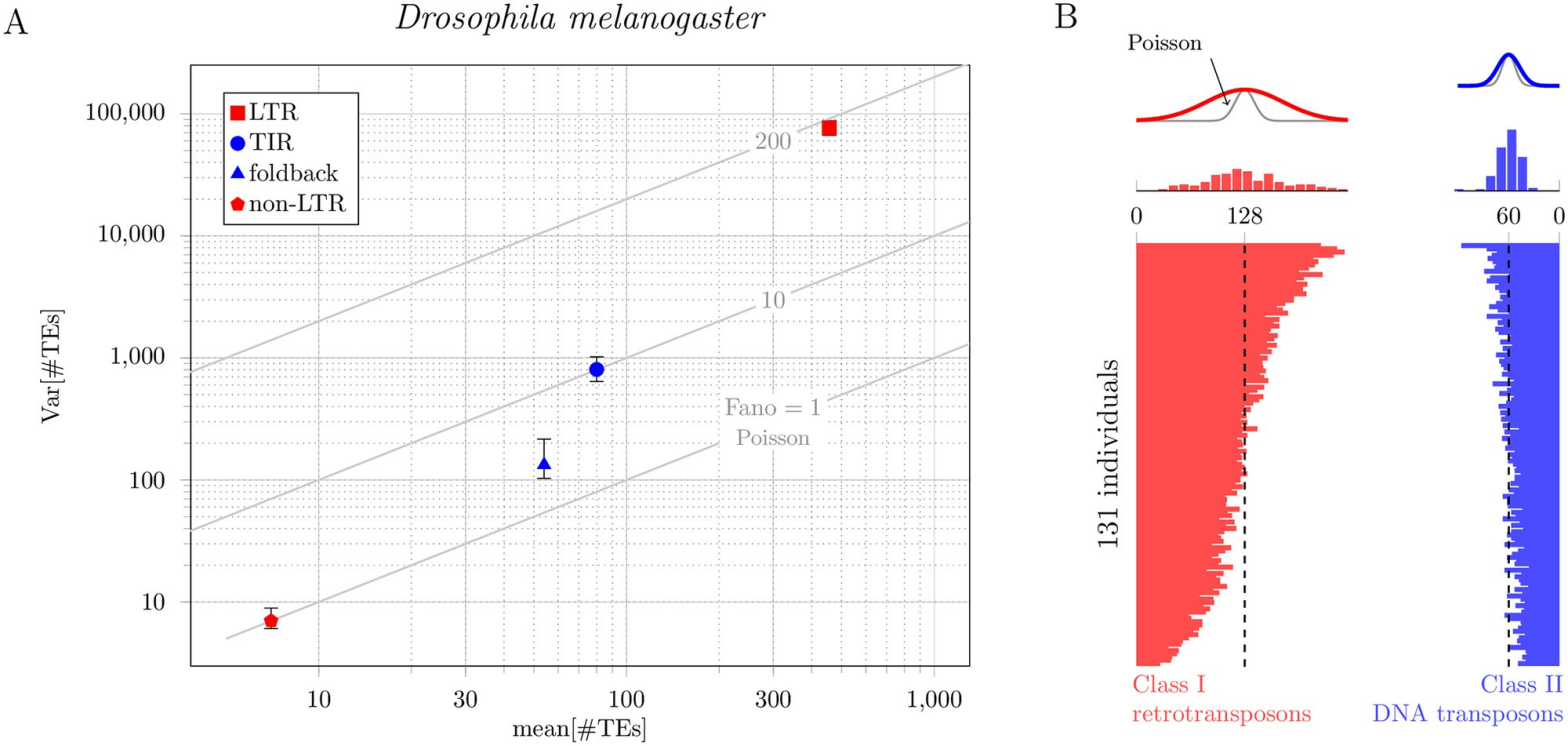
Empirically observed distributions of TE copy number in a *Drosophila melanogaster* population. A: Mean-variance plot of TE copy number for 131 individuals compared to theoretical expectation (Fano = 1, Poisson diagonal line). For each of four different families of TEs, the index of dispersion is in the range 1<Fano[n]<200. Vertical bars show 95% bootstrap confidence intervals. B: TE copy number for 131 *D. melanogaster* individuals. TE counts are separated by class (red, Class I, retrotransposon; blue, Class II, DNA transposon).

Figs 1 and 2 present analyses of two data sets, both of which demonstrate that the variance of TE load in experimentally studied populations can be far greater than would be predicted by classical models of TE population genetics. The first data set (analyzed in Fig 1) consists of whole-genome sequence data from 164 lines of *Mimulus guttatus* derived from a naturally occurring population (hundreds of thousands of individuals) in Iron Mountain, Oregon, USA [15]. To estimate TE copy numbers, we compared the coverage of each TE to the average coverage of single copy genes (see Appendix: Data Analysis for details). Although *Mimulus guttatus* is our primary interest, we also analyzed (Fig 2), for comparison, genomic DNA sequencing data from 131 lines of *Drosophila melanogaster* (derived from a large population in Raleigh, North Carolina) from the Drosophila Genetic Reference Panel [16].

Comparison of the marker locations and histograms in Figs 1 and 2 with the gray lines labelled Poisson shows that in both species, *Mimulus guttatus* and *Drosophila melanogaster*, the population distribution of TE load is *overdispersed* (the variance of TE load is greater than the mean TE load). In *D. melanogaster*, this overdispersion is greater for Class I TEs (retrotransposons) with an RNA intermediate than Class II TEs (DNA transposons) (Fig 2B). The corresponding Fano factors, given by Eq (10), are 16 and 2.7, respectively (see Table 1). Fig 1A shows that overdispersion of TE load is even more pronounced in *M. guttatus*. In this case, the Fano factors are 61 for Class I TEs (red symbols: LINE, SINE and LTR), and 646 for Class II TEs (blue symbols: CACTA, Helitron, Tcl-Mariner, PIF-Harbinger, hAT and MULE). Fig 1B shows the estimated number of Class I and II TEs in each of the 164 lines of *M. guttatus* (horizontal bar graph). In both cases, the variance (width of red and blue histograms) is far greater than the variance in classical models of TE population genetics (gray curves). Taken together, Figs 1 and 2 show that in both species (*M. guttatus* and *D. melanogaster*) and for both classes of TEs (retrotransposons and DNA transposons), the population distribution of TE load is highly *overdispersed*.

**Table 1:** Empirically observed overdispersion of TE load.

Species	TE Class	E[n]	Var[n]	Fano[n]
*M. guttatus*	I	8,082	4.9 × 10^5^	61
	II	27,559	1.8 × 10^7^	646
*D. melanogaster*	I	128	2,053	16
	II	60	164	2.7

Mean, variance, and index of dispersion (Fano factor) of the population distribution of TE load in 164 *M. guttatus* and 131 *D. melanogaster* individuals (cf. Figs 1 and 2). Class I elements (retrotransposons) proliferate in a staged manner that involves an RNA intermediate, while Class II elements (DNA transposons) do not utilize an RNA intermediate (for review see Ch. 9 of [17]).

### Overdispersion is not explained by distinct TE families

The overdispersion documented in Figs 1 and 2 cannot be explained away as a simple consequence of heterogeneous properties of distinct TE types. Consider two families of TEs with loads across individuals in the population given by the random variables **x**_1_ and **x**_2_. Denoting the mean TE loads of these families by ${\bar{n}}_{i}=E\left[x_{i}\right]$, the corresponding Fano factors are $F_{i}=\text{Var}\left[x_{i}\right]/{\bar{n}}_{i}$. If these two families were not distinguished, the observed mean load would be given by a composite count, **x** = **x**_1_ + **x**_2_, with mean $\bar{n}={\bar{n}}_{1}+{\bar{n}}_{2}$ and variance,

$$\begin{array}{ccc} \text{Var}[x] & = & \text{Var}\left[x_{1}+x_{2}\right]=\text{Var}\left[x_{1}\right]+\text{Var}\left[x_{2}\right]+2\text{Cov}\left[x_{1},x_{2}\right]\\ & = & {\bar{n}}_{1}F_{1}+{\bar{n}}_{2}F_{2}+2\text{Cov}\left[x_{1},x_{2}\right]\;. \end{array}$$ (11)

Substituting $\text{Var}\left[x_{i}\right]={\bar{n}}_{i}F_{i}$ and dividing by $\bar{n}$ gives an expression for the composite index of dispersion,

$$\begin{array}{c} F=\frac{\text{Var}[x]}{\bar{n}}=\frac{F_{1}{\bar{n}}_{1}+F_{2}{\bar{n}}_{2}}{{\bar{n}}_{1}+{\bar{n}}_{2}}+\frac{2}{\bar{n}}\text{Cov}\left[x_{1},x_{2}\right]\;. \end{array}$$

Assuming that the within-population loads for the two families of TEs are independent, the covariance will be zero ($\text{Cov}[x_{1},x_{2}]=0$). In that case, the composite Fano factor is a weighted average of Fano factors for each family,

$$\begin{array}{c} F=\frac{F_{1}{\bar{n}}_{1}+F_{2}{\bar{n}}_{2}}{{\bar{n}}_{1}+{\bar{n}}_{2}}\;, \end{array}$$

which takes values in the range min(*F*_1_, *F*_2_) ≤ *F* ≤ max(*F*_1_, *F*_2_). A similar argument allows us to conclude that for TE families with independent proliferation and excision dynamics, the dispersion of TE load that results when families are not distinguished is always *less* than the overdispersion of at least one of the TE families. When the dynamics of TE families are not independent the situation is more complicated. The composite Fano factor may either increase or decrease when families of TEs are lumped into larger groups, or split into smaller groups, depending on the mean load for each family and the correlation (positive or negative) of loads in the population (see Sec 2 of S1 Text for discussion).

### Master equation for TE population dynamics

Our modeling aims to clarify the observed overdispersion of TE load in *M. guttatus* and *D. melanogaster*, following classical TE population genetics, but with a few important modifications. Because the variance in TE load is not the result of heterogeneity in TE types (see above), our analysis will focus on a single TE family.

Let *p*_*n*_(*t*) denote the probability that a randomly sampled haploid genome (gamete) has a TE count of *n* at time *t*. Prior to considerations of selection, the model of TE population dynamics is a skip-free birth-death process with gain and loss rates denoted *g*_*n*_ and *ℓ*_*n*_. The discrete state space for haploid TE load is **n** ∈ {0, 1, 2, …, *m*} and the state-transition diagram of the stochastic process is

$$\begin{array}{c} 0\overset{g_{0}}{\underset{l_{1}}{⇌}}1\;\cdots \;n-1\overset{g_{n-1}}{\underset{l_{n}}{⇌}}n\overset{g_{n}}{\underset{l_{n+1}}{⇌}}n+1\;\cdots \;m-1\overset{g_{m-1}}{\underset{l_{m}}{⇌}}m. \end{array}$$ (12)

The master equation for this stochastic process is the following system of *m*+ 1 differential equations,

$$\begin{array}{ccc} \frac{dp_{0}}{dt} & = & -g_{0}p_{0}+\ell_{1}p_{1} \end{array}$$ (13)

$$\begin{array}{ccc} \frac{dp_{n}}{dt} & = & g_{n-1}p_{n-1}-g_{n}p_{n}-\ell_{n}p_{n}+\ell_{n+1}p_{n+1}\;\;1\leq n\leq m-1 \end{array}$$ (14)

$$\begin{array}{ccc} \frac{dp_{m}}{dt} & = & g_{m-1}p_{m-1}-\ell_{m}p_{m}\;. \end{array}$$ (15)

Each term of the master equation corresponds to gain or loss of probability for a given state. For example, Eq (12) includes the transition *n* ⇀ *n* + 1 with rate constant *g*_*n*_. This transition, which occurs at rate *g*_*n*_*p*_*n*_, results in loss of probability for state *n*; hence Eq (14), which gives the rate of change of *p*_*n*_, includes the term −*g*_*n*_*p*_*n*_ (negative). The transition *n* − 1 ⇀ *n* with rate constant *g*_*n*−1_ occurs at rate *g*_*n*−1_*p*_*n*−1_ and results in gain of probability for state *n*. The corresponding term in the *dp*_*n*_/*dt* equation is *g*_*n*−1_*p*_*n*−1_ (positive). The expected value of TE load of a randomly sampled diploid genotype is

$$\begin{array}{c} \bar{n}=E\left[n\right]=2\sum_{n=0}^{m}np_{n}=2\mu_{1}\;, \end{array}$$ (16)

where $\mu_{1}=\sum_{n=0}^{m}np_{n}$ is the mean TE load of a randomly sampled haploid gamete. By differentiating Eq (16) to obtain

$$\begin{array}{c} \frac{d\bar{n}}{dt}=2\sum_{n=0}^{m}n\frac{dp_{n}}{dt}\;, \end{array}$$ (17)

and substituting Eqs (13)–(15), the master equation formulation can be shown to be consistent with the classical approach (see Sec 3 of S1 Text).

### The master equation model predicts the variance of TE load

The dynamics of the population variance of TE load are an emergent property of the master equation model, Eqs (13)–(15). To illustrate, let us assume that TE excision occurs with a first-order rate constant. In that case, the loss rate as a function of *n* is

$$\begin{array}{c} \ell_{n}=n\nu \;. \end{array}$$ (18)

Let us further assume that the rate of gain for a single family of TEs takes the form

$$\begin{array}{c} g_{n}=nu_{n}=\left(\eta_{0}+\eta n\right)\left(1-n/m\right)\;. \end{array}$$ (19)

**Fig 3:**
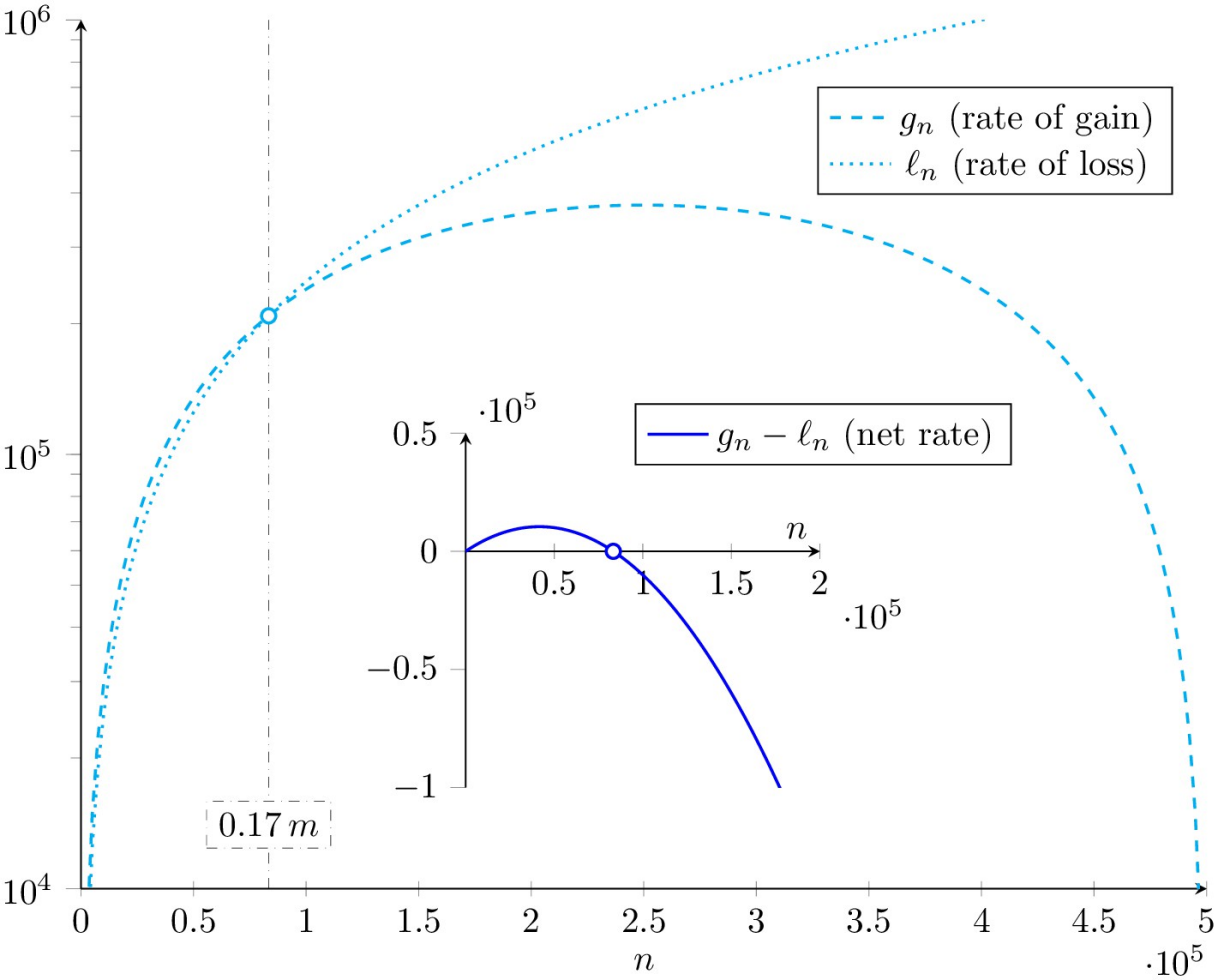
Rates of transposable element gain and loss in master equation model. Example rates of transposable element gain (*g*_*n*_, dashed) and loss (*ℓ*_*n*_, dotted) are shown cyan. These functions of TE load given by Eqs (18) and (19) intersect (balance) when 17% of the insertion sites are occupied (gray dot-dashed line). Parameters: *η*_0_ = 20, *η* = 3, *ν* = 2.5, *m* = 5 × 10^5^. The net rate of change (blue curve) is zero for a TE load of *n* = 8.3 × 10^4^ (open circle), which is on the order of that found for transposons (Class II elements) in *Mimulus* (e.g., LINE and LTR, see Fig 1B left).

In this expression, *η* is the copy-and-paste rate per transposon (a first-order rate constant characterizing proliferation of TEs), *η*_0_ is a zeroth order rate constant, *n* is the TE copy number, and *m* is the number of occupiable loci (in a haploid gamete). Fig 3 shows these TE gain and loss rates, *ℓ*_*n*_ and *g*_*n*_, as functions of *n*. Substituting these constitutive relations into Eqs (13)–(15) gives

$$\begin{array}{ccc} \frac{dp_{0}}{dt} & = & -\eta_{0}p_{0}+\nu p_{1} \end{array}$$ (20)

$$\begin{array}{ccc} \frac{dp_{n}}{dt} & = & [\eta_{0}+\eta \left(n-1\right)][1-(n-1)/m]p_{n-1}\\ & & -[\left(\eta_{0}+\eta n\right)\left(1-n/m\right)+\nu n]p_{n}\\ & & +[\nu (n+1)]p_{n+1} \end{array}$$ (21)

$$\begin{array}{ccc} \frac{dp_{m}}{dt} & = & [\eta_{0}+\eta \left(m-1\right)][1-(m-1)/m]p_{m-1}-\nu mp_{m}\;. \end{array}$$ (22)

where 1 ≤ *n* ≤ *m* − 1 in Eq (21).

**Fig 4:**
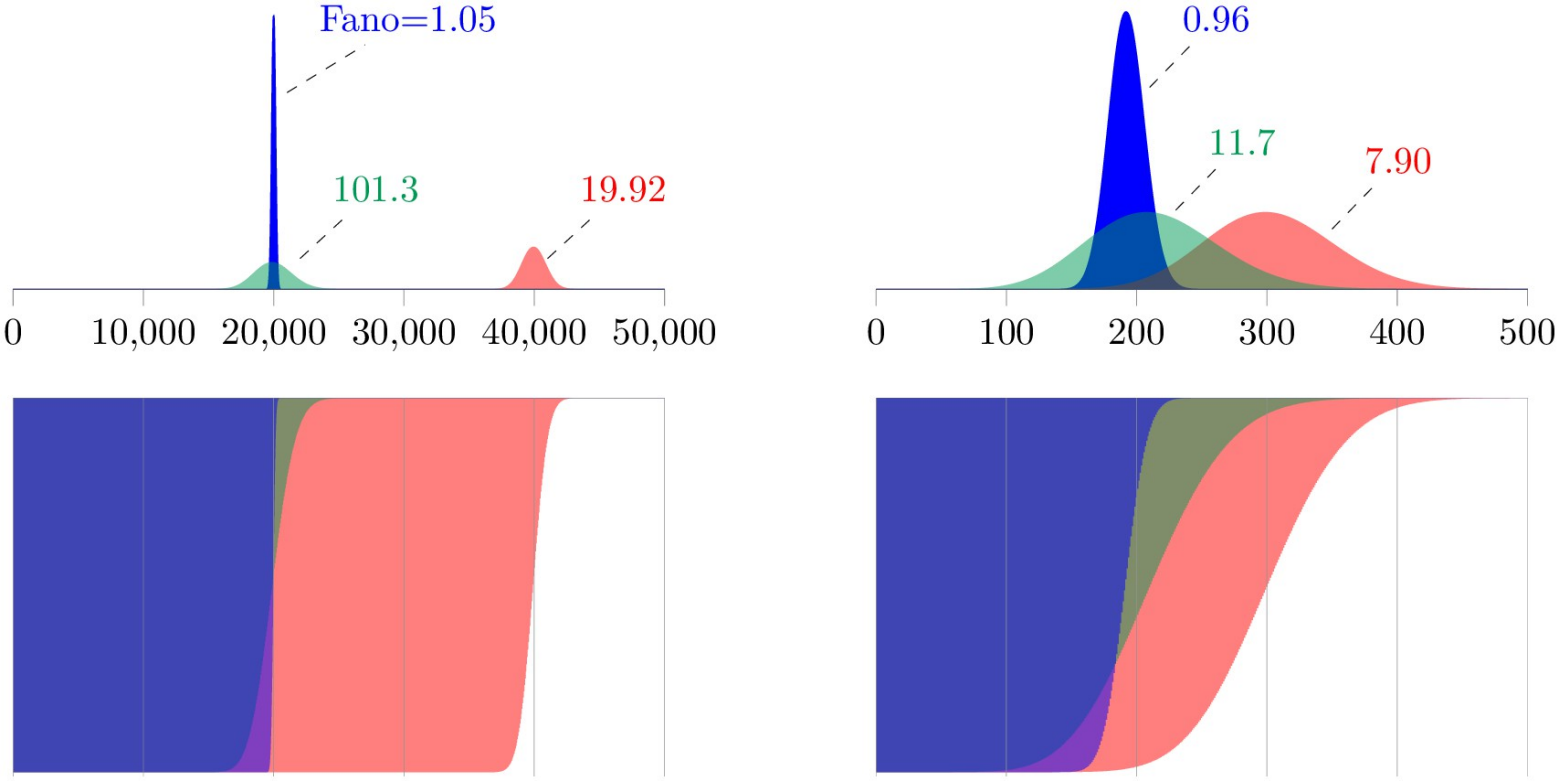
Gametic TE load calculated using the master equation model. Top: Stationary population distributions of TE load in haploid genomes calculated using the evolutionarily neutral master equation model, Eqs (20)–(22). For mean loads similar to *Mimulus* (left) and *Drosophila* (right), no overdispersion is observed in simulations absent copy-and-paste transposition (*η* = 0, Fano[n]≈1). Green and red histograms show overdispersed population distributions of TE load that are obtained when copy-and-paste transposition is included. *Mimulus* parameters: *ν* = 0.1, *m* = 10^9^; *η*_0_, *η* = 2000, 0 (blue), 200, 0.095 (red), 20, 0.099 (green). *Drosophila* parameters: *ν* = 0.1, *m* = 5000; *η*_0_, *η* = 20, 0 (blue), 1, 0.1 (green), 2, 0.1 (red). See Sec 6 of S1 Text for numerical methods.

Fig 4 shows representative numerical solutions of this master equation for the population dynamics of TE load. When the copy-and-paste rate constant is zero (*η* = 0) and occupiable loci are not limiting ($\bar{n}<<2m$), the stationary probability distribution is well-approximated by a Poisson distribution with $\text{Var}\left[n\right]\approx \bar{n}$ and Fano[n]≈1 (blue histograms). For both *Mimulus*- and *Drosophila*-like parameters, no overdispersion is observed when *η* = 0. These results should be compared to the green and red histograms, for which the copy-and-pate rate is nonzero (see caption for parameters). Notably, an increase in the copy-and-paste rate leads to significant overdispersion of the TE load for both simulated populations (Fano[n] ranging from 1 to 100).

### Moment equations for mean and variance of TE load

The previous section showed that the evolutionarily neutral master equation model provides information about the population variance of TE load that is unavailable in classical theory. Because this realism introduces complexity—Eqs (20)–(22) compared to Eq (2)—we derived ordinary differential equations (ODEs) that summarize the dynamics of the mean and variance of the population distribution of diploid TE loads predicted by the master equation. Sec 3 of S1 Text shows that the mean and variance of TE load solve the following ODEs,

$$\begin{array}{ccc} \frac{d\bar{n}}{dt} & = & 2\eta_{0}-(\nu -\eta +\frac{\eta_{0}}{m})\bar{n}-\frac{\eta }{m}(\sigma_{n}^{2}+\frac{{\bar{n}}^{2}}{2}) \end{array}$$ (23)

$$\begin{array}{ccc} \frac{d\sigma_{n}^{2}}{dt} & = & 2\eta_{0}+(\nu +\eta -\frac{\eta_{0}}{m})\bar{n}-2(\nu -\eta +\frac{\eta_{0}+\eta /2}{m})\sigma_{n}^{2}\\ & - & \frac{2\eta }{m}(\bar{n}\sigma_{n}^{2}+\frac{{\bar{n}}^{2}}{4}+E\left[{\left(n-\bar{n}\right)}^{3}\right])\;. \end{array}$$ (24)

The term $E[{\left(n-\bar{n}\right)}^{3}]$ that appears in Eq (24) is the third central moment of the within-population diploid TE load. Analysis of this system of ODEs and the third central moment is provided below.

If number of occupiable loci are not limiting ($\bar{n}<<2m$), we may take the limit of Eqs (23) and (24) as *m* → ∞ to obtain simpler equations for the mean and variance,

$$\begin{array}{ccc} \frac{d\bar{n}}{dt} & = & 2\eta_{0}-\left(\nu -\eta \right)\bar{n} \end{array}$$ (25)

$$\begin{array}{ccc} \frac{d\sigma_{n}^{2}}{dt} & = & 2\eta_{0}+\left(\nu +\eta \right)\bar{n}-2\left(\nu -\eta \right)\sigma_{n}^{2}\;. \end{array}$$ (26)

This reduced system of ODEs is linear and, for large *m*, the equation for the variance, Eq (26), does not depend on the third central moment. The steady-state solution of Eqs (25) and (26) is given by

$$\begin{array}{ccc} \bar{n} & = & \frac{2\eta_{0}}{\nu -\eta } \end{array}$$ (27)

$$\begin{array}{ccc} \sigma_{n}^{2} & = & \frac{2\eta_{0}\nu }{{\left(\nu -\eta \right)}^{2}}=\frac{\nu \bar{n}}{\nu -\eta } \end{array}$$ (28)

is physical provided *ν* > *η*, that is, when *m* is large, the rate of excision *ν* must be greater than the copy-and-past rate constant *η* for a biologically meaningful solution with $\bar{n}\geq 0$ (mean TE load must be non-negative). This steady state is stable because the Jacobian of Eqs (25) and (26), given by the 2 × 2 matrix with entries *J*_11_ = −(*ν* − *η*), *J*_12_ = 0, *J*_21_ = *ν* + *η*, *J*_22_ = −2(*ν* − *η*), has real valued eigenvalues λ_+_ = −(*ν* − *η*) < 0 and λ_−_ = 2λ_+_ < 0.

The values for the steady-state mean and variance of TE load given by Eqs (27) and (28) correspond to the following index of dispersion,

$$\begin{array}{c} \text{Fano}\left[n\right]=\frac{\sigma_{n}^{2}}{\bar{n}}=\frac{\nu }{\nu -\eta }\;. \end{array}$$ (29)

Notably, the condition for a stable steady state (*ν* > *η*) implies an index of dispersion greater than unity (Fano[n]>1) for any nonzero copy-and-paste rate constant (*η* > 0). For this reason, we conclude that *copy-and-paste proliferation dynamics will result in an overdispersed steady-state population distribution of TE loads provided the number of occupiable loci are not limiting* ($\bar{n}<<2m$). Further analysis of the moment equations, Eqs (23) and (24), shows that overdispersion will not occur in the absence of copy-and-paste dynamics (see the *η* = 0 case in Table 2).

**Table 2:** Mean and variance of TE load in the absence of selection.

Limit	$E\left[n\right]=\bar{n}$	$\text{Var}\left[n\right]=\sigma_{n}^{2}$	Fano[n]=Var[n]/E[n]
*η* = 0	$\frac{2m\;\eta_{0}/\nu }{m+\eta_{0}/\nu }$	$\frac{2m^{2}\;\eta_{0}/\nu }{{\left(m+\eta_{0}/\nu \right)}^{2}}$	$\frac{m}{m+\eta_{0}/\nu }$
*ν* > *η*, *m* → ∞	$\frac{2\eta_{0}}{\nu -\eta }$	$\frac{2\eta_{0}\nu }{{\left(\nu -\eta \right)}^{2}}$	$\frac{\nu }{\nu -\eta }$

The evolutionarily neutral moment equations, Eqs (23) and (24), make predictions for the mean and variance of TE load in various limits (see Secs 3.2–3.3 of S1 Text). The influence of selection on overdispersion can be understood by comparison.

**Fig 5:**
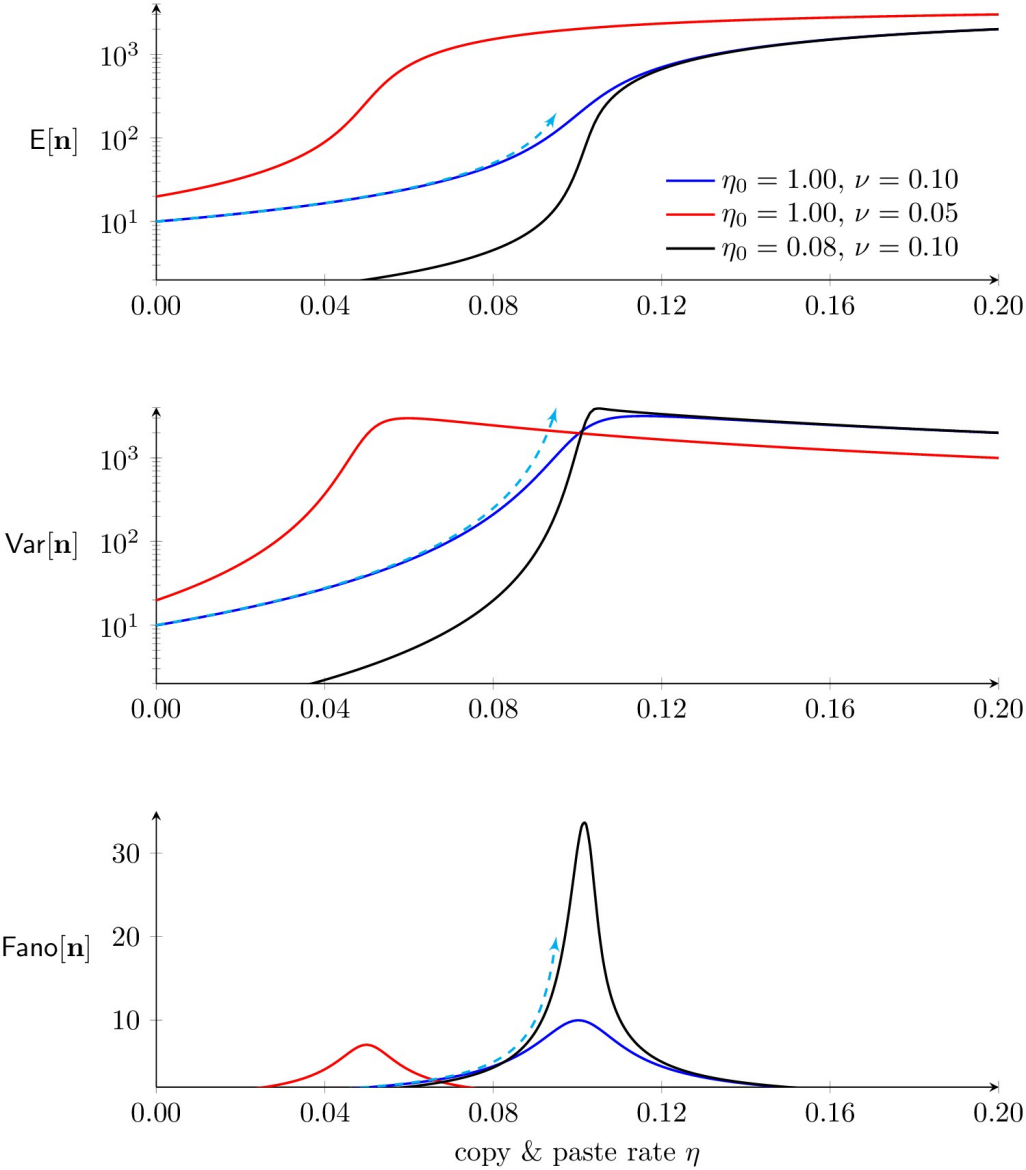
Overdispersion of TE load depends on the copy-and-paste rate (*η*). Parameter studies of the neutral master equation model showing the mean ($\bar{n}$), variance ($\sigma_{n}^{2}$), and index of dispersion (Fano[n]) of within-population TE load as a function of the copy-and-paste rate constant (*η*). Parameters: *m* = 4 × 10^3^ and as in legend. Cyan curves indicate analytical approximations using *ν* = 0.1 that are valid in the limit as *m* → ∞ (see Table 2). These approximations are most accurate for small *η*/*ν* and diverge as *η* approaches *ν* from below (cyan arrowheads). These calculations were accelerated using a Fokker-Planck approximation to Eqs (20)–(22) (see Sec 6 in S1 Text).

This preliminary analysis of an evolutionarily neutral master equation for TE proliferation, Eqs (20)–(22), indicates that *a nonzero copy-and-paste rate may lead to an overdispersed population distribution of TE load*, as in Eq (29). That is, copy-and-paste TE dynamics is one possible explanation for our empirical observations of overdispersed TE counts (Figs 1 and 2). Furthermore, this analysis predicts that a large index of dispersion may be a consequence of balanced dynamics of TE gain and loss, that is, Fano[n]→∞ as *ν* decreases to *η* in Eq (29). While the divergence in the analytical result is an artifact of taking the *m* → ∞ limit, a parameter study of the master equation model (Fig 5) confirms that overdispersion is most pronounced (Fano[n] maximized) when *m* is large and the dynamics of TE gain and loss are approximately balanced (*η* ≈ *ν*).

### Influence of selection on overdispersion

To investigate the effect of purifying selection on the population variance of TE load, we assume a selection coefficient (*w*_*n*_) that depends on total diploid TE load (*n*) with *dw*_*n*_/*dn* < 0 (higher load is less viable). For concreteness, let

$$\begin{array}{c} w_{n}={\left(1-s\right)}^{\ell }\;\text{for}\;0\leq s<<1\;, \end{array}$$ (30)

where *s* is the strength of selection against TE load. When the neutral model, Eqs (20)–(22), is modified to include selection, the master equation becomes

$$\begin{array}{ccc} \frac{dp_{n}}{dt} & = & \alpha \left(p_{n}^{\prime }-p_{n}\right)+[\eta_{0}+\eta \left(n-1\right)][1-(n-1)/m]p_{n-1}\\ & & -[\left(\eta_{0}+\eta n\right)\left(1-n/m\right)+\nu n]p_{n}+\nu \left(n+1\right)p_{n+1}\;. \end{array}$$ (31)

for 1 ≤ *n* ≤ *m*. The first term in this expression represents each load probability *p*_*n*_ relaxing to a target probability $p_{n}^{\prime }$ given by

$$\begin{array}{c} p_{n}^{\prime }=\frac{p_{n}\sum_{j}w_{n+j}p_{j}}{\sum_{i}p_{i}\sum_{j}w_{i+j}p_{j}}\;\;0\leq i,j\leq m\;, \end{array}$$ (32)

where *w*_*i*+*j*_ = (1 − *s*)^*i*+*j*^. The equations for for *dp*_0_/*dt* and *dp*_*m*_/*dt* have fewer gain/loss terms than Eq (31), but are analogous to Eqs (20)–(22). The parameter *α* that occurs in Eq (31) is the inverse of the generation time. The quantity $\bar{w}=\sum_{i}p_{i}\sum_{j}w_{i+j}p_{j}$ is the mean fitness under the assumption of random mating [18].

**Fig 6:**
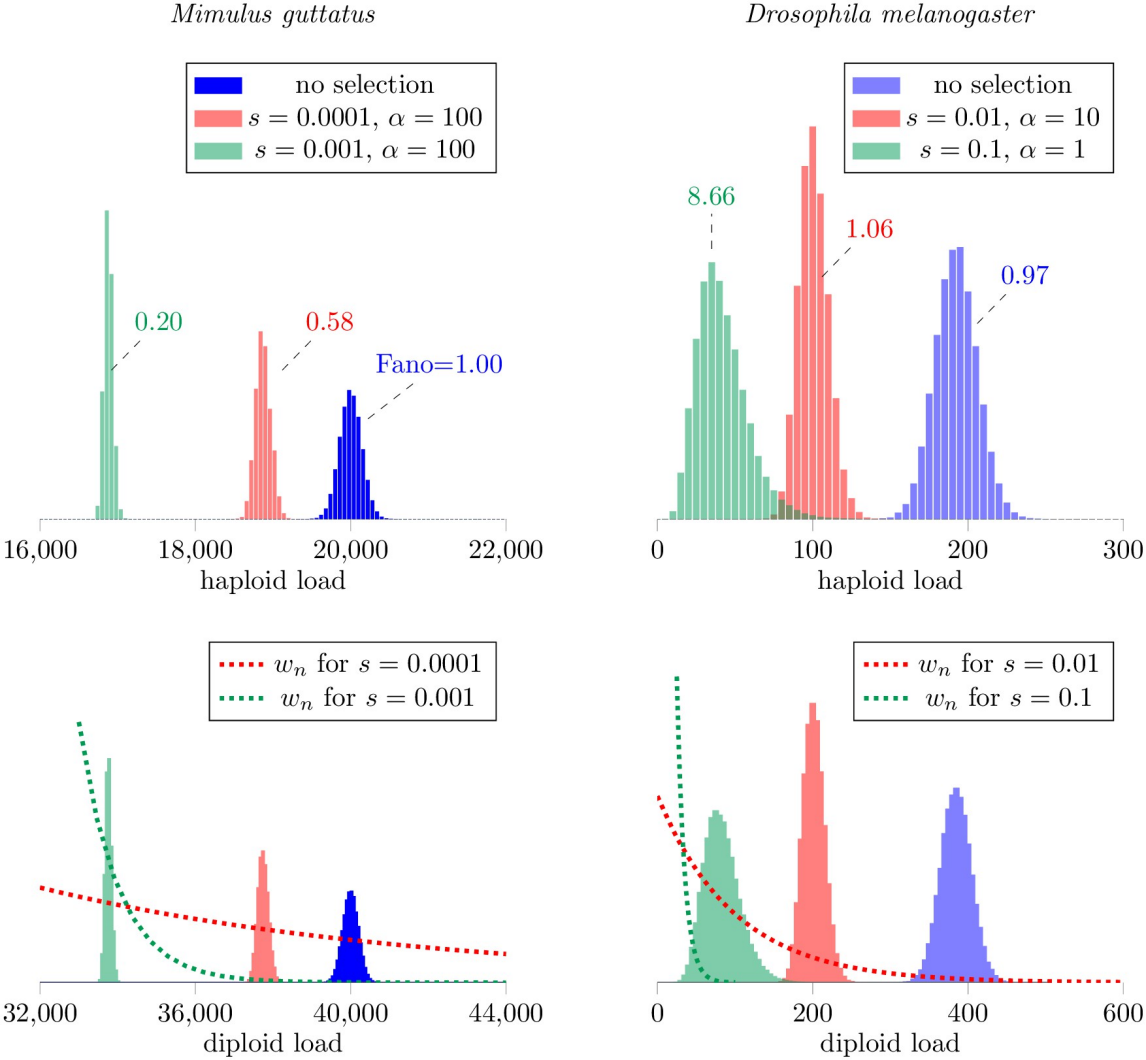
Effect of selection on the distribution of TE load. Stationary population distributions of TE abundance with and without selection predicted by the master equation model, Eqs (31) and (32). Parameters: selection coefficient *s* as in legends. *Mimulus*: *ν* = 0.1, *m* = 10^9^; *η*_0_, *η* = 2000, 0 (blue), 200, 0.095 (red), 20, 0.099 (green). *Drosophila*: *ν* = 0.1, *m* = 5000; *η*_0_, *η* = 20, 0 (blue), 1, 0.1 (green), 2, 0.1 (red).

Fig 6 shows steady-state distributions of haploid (top row) and diploid (bottom) TE loads calculated using Eq (31) both with and without of selection on diploid load. As expected, the effect of weak selection (red and green histograms) is to decrease the TE load in the population as compared to the neutral model (blue histograms). This decrease in mean TE load occurs for a wide range of generation times (1/*α*) and selection coefficients (*s*). More important (and less obvious) is the impact of selection on the variance of TE load and overdispersion. Using *Drosophila* parameters, Fig 6 (top right) shows an example simulation (green histogram) in which selection leads to increased dispersion (the Fano factor increases from 1 to 8.66). However, in a second case (red histogram), selection increases the index of dispersion only slightly (to a Fano factor of 1.06). Notably, in three representative simulations using *Mimulus* parameters, selection does not increase the dispersion of TE load (Fig 6, left). This observation is consistent with the moment-based analysis presented in the following section.

### Moment equations with selection

For a deeper understanding of the impact of selection on the distribution of TE load in a population, one may begin with Eqs (31) and (32) and derive the dynamics of the mean and variance of TE load under the action of simple selection functions. For example, in the limit of weak selection 0 < *s* < < 1, Eq (30) is well-approximated by *w*_*n*_ = 1 − *sn*. In this case, as derived in Sec 4 of S1 Text, the dynamics of the mean and variance of TE load solve

$$\begin{array}{ccc} \frac{d\bar{n}}{dt} & = & -\frac{\alpha s}{1-s\bar{n}}\cdot \sigma_{n}^{2}+2\eta_{0}-(\nu -\eta +\frac{\eta_{0}}{m})\bar{n}-\frac{\eta }{m}(\sigma_{n}^{2}+\frac{{\bar{n}}^{2}}{2}) \end{array}$$ (33)

$$\begin{array}{ccc} \frac{d\sigma_{n}^{2}}{dt} & = & -\frac{\alpha s}{1-s\bar{n}}\cdot E\left[{\left(n-\bar{n}\right)}^{3}\right]+2\eta_{0}+(\nu +\eta -\frac{\eta_{0}}{m})\bar{n}-2(\nu -\eta +\frac{\eta_{0}+\eta /2}{m})\sigma_{n}^{2}\\ & - & \frac{2\eta }{m}(\bar{n}\sigma_{n}^{2}+\frac{{\bar{n}}^{2}}{4}+E\left[{\left(n-\bar{n}\right)}^{3}\right])\;. \end{array}$$ (34)

These ODEs may be compared to the moment equations for the neutral model, Eqs (25) and (26). As expected, the influence of selection on the mean TE load is proportional to the population variance through the factor $-\alpha s\sigma_{n}^{2}/\left(1-s\bar{n}\right)$ in Eq (33). Similarly, the influence of selection on the population variance is proportional to the third central moment of the diploid load through the factor $-\alpha sE\left[{\left(n-\bar{n}\right)}^{3}\right]/\left(1-s\bar{n}\right)$ in Eq (34). In both cases, the quantity $1-s\bar{n}$ is the mean fitness of the population, i.e., $\bar{w}=E\left[w_{n}\right]=E\left[1-sn\right]=1-s\bar{n}$.

Under the assumption that the mean TE load is much smaller than the number of loci ($\bar{n}<<2m$), we may simplify Eqs (33) and (34) by taking the limit *m* → ∞,

$$\begin{array}{ccc} \frac{d\bar{n}}{dt} & = & -\frac{\alpha s}{1-s\bar{n}}\cdot \sigma_{n}^{2}+2\eta_{0}-\left(\nu -\eta \right)\bar{n} \end{array}$$ (35)

$$\begin{array}{ccc} \frac{d\sigma_{n}^{2}}{dt} & = & -\frac{\alpha s}{1-s\bar{n}}\cdot E\left[{\left(n-\bar{n}\right)}^{3}\right]+2\eta_{0}+\left(\nu +\eta \right)\bar{n}-2\left(\nu -\eta \right)\sigma_{n}^{2}\;. \end{array}$$ (36)

Setting the left side of Eq (35) to zero, we observe that the steady-state mean and variance are related as follows,

$$\begin{array}{c} \bar{n}=\frac{2\eta_{0}}{\nu -\eta }-\frac{\alpha s}{1-s\bar{n}}\cdot \frac{\sigma_{n}^{2}}{\nu -\eta }=\frac{2\eta_{0}}{\nu -\eta }[1-\frac{\alpha s}{1-s\bar{n}}\cdot \frac{\sigma_{n}^{2}}{2\eta_{0}}]\;. \end{array}$$ (37)

Comparing this expression to Eq (27) and noting that the variance is nonnegative ($\sigma_{n}^{2}\geq 0$), we see that weak selection decreases the mean TE load in the population as compared to the neutral model (as expected). Similar analysis of Eq (36) shows how selection may impact the variance of TE load and, consequently, the index of overdispersion. Setting the left side of Eq (36) to zero and solving for the steady-state variance, gives

$$\begin{array}{c} \sigma_{n}^{2}[1+\frac{\alpha s}{1-s\bar{n}}\cdot \frac{\nu +\eta }{2{\left(\nu -\eta \right)}^{2}}]=\frac{2\eta_{0}\nu }{{\left(\nu -\eta \right)}^{2}}-\frac{\alpha s}{1-s\bar{n}}\cdot \frac{E[{\left(n-\bar{n}\right)}^{3}]}{2(\nu -\eta )}\;, \end{array}$$ (38)

where the first term on the right side, 2*η*_0_*ν*/(*ν* − *η*)^2^, is the variance of TE load in the absence of selection. Eq (38) shows weak selection can decrease or increase the population variance of TE load, depending on the sign of the third central moment ($E[{\left(n-\bar{n}\right)}^{3}]$), consistent with master equation simulations (Fig 6).

### Moment closure and the ($\bar{n}$, $\sigma_{n}^{2}$) phase plane

In their current form, the moment equations with selection, Eqs (33) and (34), are an open system of ODEs. That is, the equation for the variance ($\sigma_{n}^{2}$) depends on $E[{\left(n-\bar{n}\right)}^{3}]$, the unknown third central moment. As discussed in Sec 5 of S1 Text, an applicable moment closure technique assumes that the third central moment of the diploid load is algebraic function of the mean and variance,

$$\begin{array}{c} E\left[{\left(n-\bar{n}\right)}^{3}\right]=\psi \left(\bar{n},\sigma_{n}^{2}\right)\;. \end{array}$$

We investigated two possibilities for this function based on the properties of the beta-binomial and negative binomial distributions. The beta-binomial moment closure, derived in Sec 5.3 of S1 Text, is a complicated expression involving the mean, variance, and number of loci *m*,

$$\begin{array}{c} \psi_{BB}\left(\bar{n},\sigma_{n}^{2}\right)=\sigma^{2}\frac{\left(m-\bar{n}\right)\left({\bar{n}}^{2}-2m\bar{n}-2\sigma^{2}+4m\sigma^{2}\right)}{m\bar{n}\left(2m-\bar{n}-4\right)+2m\sigma^{2}+2{\bar{n}}^{2}}\;. \end{array}$$ (39)

Moment closure motivated by the properties of the negative binomial distribution results in a simpler expression that does not involve the number of loci *m*,

$$\begin{array}{ccc} \psi_{NB}\left(\bar{n},\sigma_{n}^{2}\right) & = & \sigma_{n}^{2}(\frac{2\sigma_{n}^{2}-\bar{n}}{\bar{n}})\;. \end{array}$$ (40)

Although the beta-binomial closure given by Eq (39) is arguably a better approximation, it does not perform markedly better than the negative binomial closure, Eq (40), as assessed through comparison of moment ODE and master equation simulations (not shown). In the analysis that follows, we use the negative binomial closure, motivated by its simplicity and the fact the two expressions coincide when the number of loci are not limiting (*ψ*_*BB*_ → *ψ*_*NB*_ as *m* → ∞).

Substituting Eq (40) into Eqs (33) and (34) gives a closed system of ODEs for the mean and variance of diploid load under the influence of selection:

$$\begin{array}{ccc} \frac{d\bar{n}}{dt} & = & -\frac{\alpha s}{1-s\bar{n}}\cdot \sigma_{n}^{2}+2\eta_{0}-(\nu -\eta +\frac{\eta_{0}}{m})\bar{n}-\frac{\eta }{m}(\sigma_{n}^{2}+\frac{{\bar{n}}^{2}}{2}) \end{array}$$ (41)

$$\begin{array}{ccc} \frac{d\sigma_{n}^{2}}{dt} & = & -\frac{\alpha s}{1-s\bar{n}}\cdot \sigma_{n}^{2}(\frac{2\sigma_{n}^{2}-\bar{n}}{\bar{n}})+2\eta_{0}+(\nu +\eta -\frac{\eta_{0}}{m})\bar{n}-2(\nu -\eta +\frac{\eta_{0}+\eta /2}{m})\sigma_{n}^{2}\\ & - & \frac{2\eta }{m}[\bar{n}\sigma_{n}^{2}+\frac{{\bar{n}}^{2}}{4}+\sigma_{n}^{2}(\frac{2\sigma_{n}^{2}-\bar{n}}{\bar{n}})]\;. \end{array}$$ (42)

**Fig 7:**
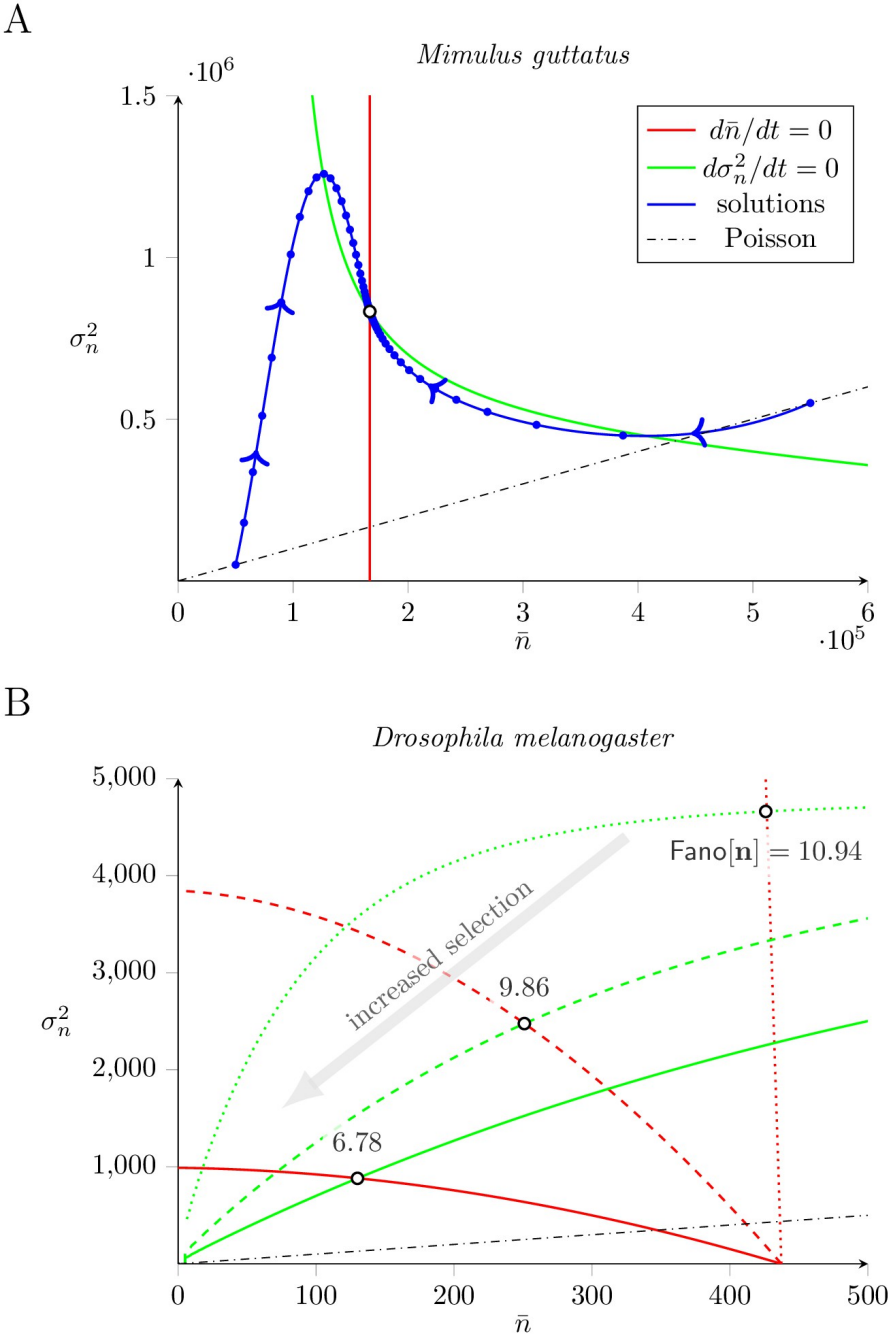
The phase plane for the dynamics of TE load. The dynamics of the mean (*n*) and variance ($\sigma_{n}^{2}$) of TE load predicted by the moment equations, Eqs (41) and (42)), can be understood through phase plane analysis. The red and green curves are the nullclines for the mean and variance, respectively, with intersection corresponding to the steady state (open circle). The blue trajectories show the dynamics of equilibration. A: Mean loads similar to *Mimulus*. Parameters: *ν* = 2.5, *m* = 5 × 10^5^, *η*_0_ = 20 and *η* = 3 with no selection (*α* = 0, *s* = 0). B: Mean loads similar to *Drosophila*. Parameters: *ν* = 0.1, *m* = 5000, *η*_0_ = 1 and *η* = 0.1. Dotted nullclines: no selection. Dashed: *s* = 10^−4^, *α* = 5. Solid: *s* = 10^−4^, *α* = 20. Note that increased selection on TE load (gray arrow) decreases the index of dispersion (Fano[n]).

Fig 7A presents a representative ($n,\sigma_{n}^{2}$) phase plane for the dynamics of the mean and variance of TE load predicted by Eqs (41) and (42). The red and green lines are the nullclines for the mean and variance, respectively, with intersection corresponding to the steady state. This calculation uses parameters resulting in a steady-state TE load similar to our empirical observations of *M. guttatus* (counts on the order of 10^5^). The moment equations predict a steady state that is located far above the broken black line denoting $\sigma_{n}^{2}=n$ and Fano factor of 1. The blue curves show two solutions, obtained by numerically integrating Eqs (41) and (42), that use initial conditions for which the population variance is equal to the mean. Interestingly, the resulting dynamics of TE load can include a transient phase in which the index of dispersion is far greater or less than the steady-state value.

Fig 7B shows how the nullclines for the mean and variance of TE load depend on the strength of selection in three cases with parameters corresopnding to TE loads similar to *D. melanogaster* (counts on the order of 100). As the strength of selection increases, both the mean and variance of TE load decrease, in such a manner that the index of dispersion (Fano[n]) also decreases.

Although the model obtained by moment closure and the phase plane analysis of Fig 7 does not assume $\bar{n}<<2m$, we may consider Eqs (41) and (42) in the limit as *m* → ∞,

$$\begin{array}{ccc} \frac{d\bar{n}}{dt} & = & -\frac{\alpha s}{1-s\bar{n}}\cdot \sigma_{n}^{2}+2\eta_{0}-\left(\nu -\eta \right)\bar{n}\\ \frac{d\sigma_{n}^{2}}{dt} & = & -\frac{\alpha s}{1-s\bar{n}}\cdot \sigma_{n}^{2}(\frac{2\sigma_{n}^{2}-\bar{n}}{\bar{n}})+2\eta_{0}+\left(\nu +\eta \right)\bar{n}-2\left(\nu -\eta \right)\sigma_{n}^{2}\;. \end{array}$$

Setting the left sides of Eqs (41) and (42) to zero, and assuming weak selection (0 ≤ *s* < < 1), we can derive first-order accurate asymptotic expressions for the steady-state mean and variance,

$$\begin{array}{ccc} \bar{n} & \approx & \frac{2\eta_{0}}{\nu -\eta }[1-\alpha s\frac{\nu }{{\left(\nu -\eta \right)}^{2}}] \end{array}$$ (43)

$$\begin{array}{ccc} \sigma_{n}^{2} & \approx & \frac{2\nu \eta_{0}}{{\left(\nu -\eta \right)}^{2}}\left[1-\alpha s\frac{\left(\nu +\eta \right)}{{\left(\nu -\eta \right)}^{2}}\right]\;. \end{array}$$ (44)

Because *v*/(*ν* − *η*)^2^ > 0, this expression indicates that weak selection decreases the mean TE load, consistent with our intuition. Similarly, the factor (*ν* + *η*)/(*ν* − *η*)^2^ is positive, allowing us to conclude that weak selection decreases the population variance when *m* is large. As for the index of dispersion, this analysis indicates that under weak selection the Fano factor is

$$\begin{array}{c} \frac{\sigma_{n}^{2}}{\bar{n}}\approx \frac{\nu }{\nu -\eta }[1-\alpha s\frac{\eta }{{\left(\nu -\eta \right)}^{2}}]\;. \end{array}$$ (45)

**Fig 8:**
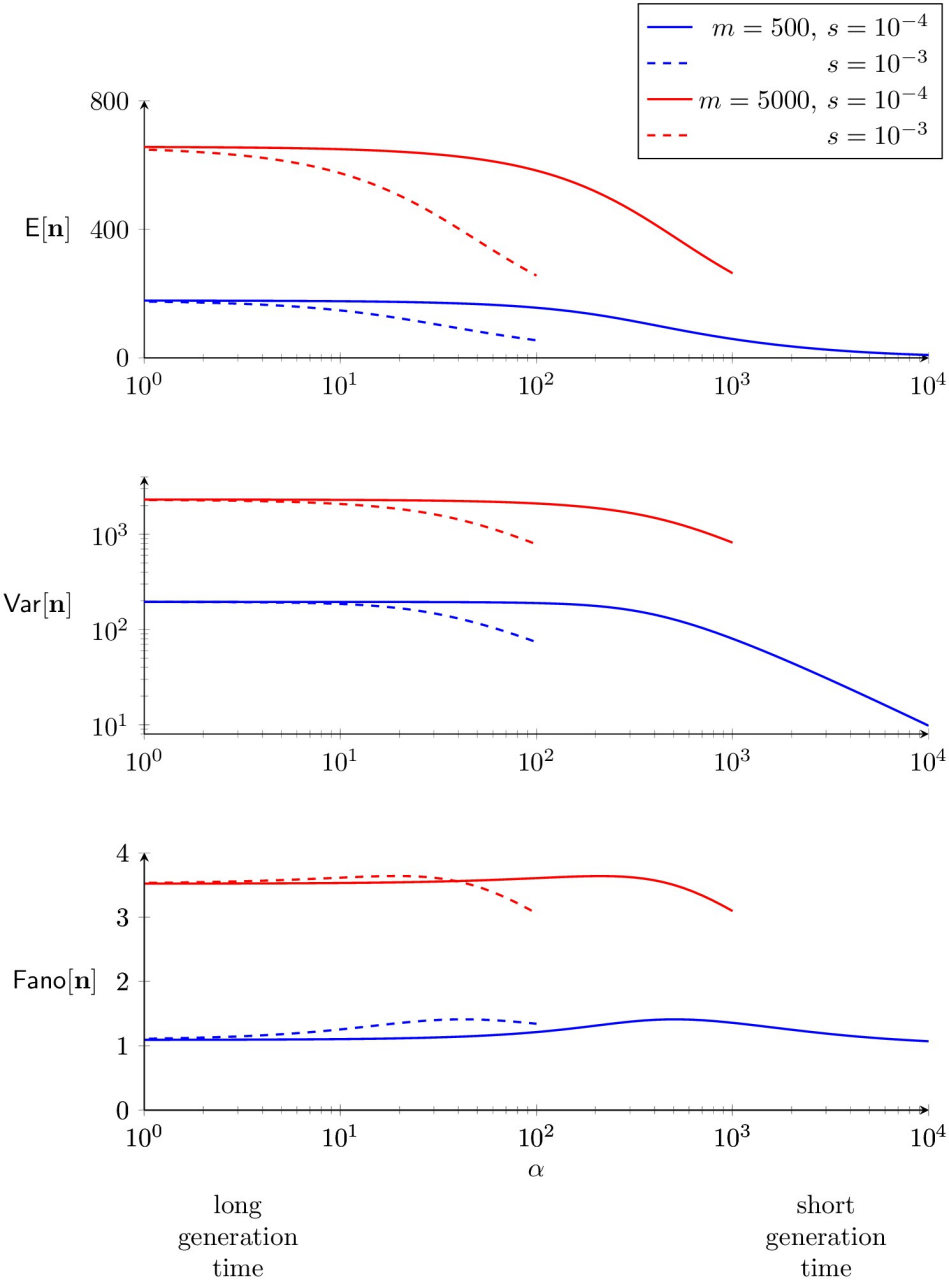
Statistics of TE load for weak selection depends on generation time. The moment equations derived under the assumption of weak selection, Eqs (33) and (34), with beta-binomial moment closure, Eq (39), enabled these parameter studies of the mean, variance, and dispersion of TE load as a function of generation time (1/*α*). Parameters: *ν* = 0.1, *η*_0_ = 10, *η* = 0.1, and as in legend.

Because *η*/(*ν* − *η*)^2^ is positive any nonzero copy-and-paste rate (*η* > 0), we conclude that the Fano factor is also expected to decrease, because weak selection causes the within-population variance of TE load to decrease more than the mean. This conclusion—i.e., selection on diploid TE load is unlikely to be responsible for overdispersion—is consistent with the numerical parameter studies summarized in Fig 8 that were enabled by the moment equations with selection, Eqs (33) and (34), and beta-binomial moment closure, Eq (39).

## Discussion

Although mathematical modeling has informed our understanding of the population genetics of transposable elements (TEs) for several decades, classical theory has emphasized analytical results that assume a binomial distribution of TE loads (see Introduction). Because the variance of a binomial distribution is less than or equal to its mean, the classical theory effectively assumes that the population distribution of TE loads are underdispersed (Fano[n]≤1).

In an empirical analysis of TE copy number in two natural populations (*M. guttatus* and *D. melanogaster*), we found—in both cases—that the population distribution of TE loads was dramatically overdispersed (Table 1, Figs 1 and 2). Because the classical theory of TE population genetics is not applicable to this situation, we extended this theory and explored mechanisms that may be responsible for observed overdispersion. The master equation model presented here predicts the entire distribution function of TE loads, and from this distribution we calculate the mean, variance, and index of dispersion as a function of model parameters.

Prior to considerations of selection, the parameters of the neutral model encode assumptions regarding the dynamics of TE proliferation (e.g., copy-and-paste and excision rate constants) as well as an estimate of the maximum number of loci that may be occupied by TEs. Using parameter sets that yield TE counts in empirically observed ranges (tens of thousands for *M. guttatus*, hundreds for *D. melanogaster*), we found—in both cases—that copy-and-paste TE proliferation dynamics often resulted in overdispersed TE loads (Fig 4). Moment-based analysis of the neutral model suggests that overdispersed population distributions are to be expected when the copy-and-paste transposition rate constant (*η*) and excision rate constant (*ν*) are approximately balanced (i.e., *η* ≈ *ν*, see Fig 5 and Table 1).

Next, we extended the master equation model to include purifying selection on TE load. For a parameter set corresponding to *M. guttatus*, selection decreased the mean and variance of TE load and, because the variance decreased more than the mean, the index of dispersion also decreased (Fig 6, left). For a parameter set corresponding to *D. melanogaster*, we found that purifying selection, when sufficiently strong, may lead to an increased index of dispersion of TE load (Fig 6, right). In both *M. guttatus* and *D. melanogaster* parameter regimes, our simulations (Fig 8) and analysis, Eqs (43)–(45), agree that *weak* purifying selection decreases both the mean and variance of TE load in such a way that the index of dispersion is unchanged or slightly increases. Moment-based analysis of the master equation confirmed that weak selection usually has the effect of decreasing the index of dispersion (Fig 7B).

It has not escaped our notice that the dynamics of mean and variance of TE load given by Eqs (41) and (42) could, at least in principle, make predictions for a longitudinal study of TE demographics. A conspicuous aspect of some trajectories in the ($n,\sigma_{n}^{2}$) phase plane is a transient phase of elevated dispersion prior to equilibration (i.e., proliferation-selection balance). Notably, this transient elevated dispersion is observed when the initial mean TE load is *less* than its equilibrium value (the concave down solution shown in Fig 7A is an example). Although beyond the scope of this paper, the transposition burst phenomenon [6–8] could be analyzed from the perspective of non-equilibrium dynamics of ($n,\sigma_{n}^{2}$) subsequent to an increase in the proliferation rate of a TE family.

### Comparison of *M. guttatus* and *D. melanogaster*

Class I elements (retrotransposons) proliferate in a staged manner that involves an RNA intermediate, while Class II elements (DNA transposons) do not utilize an RNA intermediate [19]. In our empirical analysis of TE load in *D. melanogaster*, we compared these two broad classes of TEs. We found that retrotransposons were 6-fold more highly overdispersed than DNA transposons (see Table 1 and Fig 2B). Conversely, our empirical analysis of TE load in *M. guttatus* shows that, in this natural population, DNA transposons are far more overdispersed than retrotransposons. These contrasting empirical results from *M. guttatus* and *D. melanogaster* suggest that it is the effective, and perhaps context-dependent, copy-and-past rate (*η*) of a TE family—as opposed to the mobility mechanism or TE class distinction—that is most relevant to the distribution of within-population TE load.

### Limitations of the model

The mathematical modeling presented here extends the classical theory of TE population genetics in several ways. Most importantly, in both the master equation and moment-based simulations, the relationship between the population variance and mean is a *prediction* of the model (as opposed to a modeling assumption, as in classical theory). This feature of the model enables parameter studies exploring how the dynamics of TE proliferation and purifying selection influence the dispersion of within-population TE loads.

One limitation of our model is the harsh (but common) assumption that selection acts on overall TE load [9–13]. This choice is consistent with the finding that most TE insertions have negative fitness consequences and are located outside of genes [20–23]. On the other hand, many TEs are located in heterochromatic regions of the genome. It is unlikely that these large masses of TEs have fitness consequences comparable to TEs that are proximal to genes. In future work, our model could be extended to include variability in the selective cost of TE insertions, inactivating mutations that lead to nonautonomous TEs, dead-on-arrival TE insertion, and other phenomena that, for simplicity, were not included in this study.

Arguably, the most significant limitation of the model is that the dynamics of recombination are not represented. Indeed, the population distribution of TE load is modeled without any representation of the location of TEs within the genome. To the extent that recombination promotes linkage equilibrium, one expects that recombination will decrease the dispersion of TE load and, consequently, this aspect of recombination dynamics is unlikely to be responsible for empirically observed overdispersion. We recommend interpreting the master equation and moment-based models as representations of the dynamics of a single linkage class of TEs, with the tacit understanding that the index of dispersion for a genome composed of multiple linkages classes will be less than the model prediction. Admittedly, this viewpoint does not account for the fact that recombination is less frequent in regions of the genome that have a high density of TEs. Recombination hotspots exist in *M. guttatus* that may impact patterns of TE inheritance and population variance [24]. However, studying the influence of density-dependent recombination on the dispersion of TE load is beyond the scope of this paper, as it would require a modeling framework that is explicitly spatial.

We note that events involving the loss or gain of multiple TEs (as could occur via ectopic recombination or other mechanisms) are expected to contribute to overdispersion. To see this, consider a master equation simulation in which the gain and loss of TEs occurs in blocks of size *b*. If there is no other change to the model, we may reinterpret the random variable **n** as the number of blocks of TEs in a randomly sampled diploid genome. In that case, the mean and variance of TE count are increased by a factor of *b* and *b*^2^, respectively. The Fano factor, given by the ratio of variance to mean, increases by a factor of *b*,

$$\text{Fano}\left[bn\right]=\frac{\text{Var}[bn]}{E[bn]}=\frac{b^{2}\text{Var}\left[n\right]}{bE[n]}=b\text{Fano}\left[n\right]\;.$$

This scaling implies that block-wise inheritance of TEs is expected to increase the index of dispersion by a factor proportional to a representative block size. This intriguing and relatively simple explanation for empirically observed overdispersion could be studied using an explicitly spatial model of TE population genetics, preferably one that includes a mechanistic account of ectopic recombination and perhaps other genome rearrangements.

## Appendix: Data analysis

Figs 1 and 2 present analyses of two data sets, both of which indicate that the variance of TE load in experimentally studied populations can be far greater than would be predicted by classical models of TE population genetics. The first data set (analyzed in Fig 1) consists of whole-genome sequence data from 164 lines of *Mimulus guttatus* derived from a naturally occurring population (hundreds of thousands of individuals) in Iron Mountain, Oregon, USA [15]. To estimate TE copy numbers, we compared the coverage of each TE to the average coverage of genes understood to be present in single copy. These short reads were first aligned to a composite genomic database consisting of *M. guttatus* coding sequences, the mitochondrial genome, *M. luteus* chloroplast, and a file of approximately 1400 TE sequences [24, 25]. The *M. luteus* chloroplast genome was used because it was completely assembled, *M. luteus* is closely related to *M. guttatus*, and chloroplast sequences evolve slowly making this a reasonable reference [26]. Next, the whole genome sequencing data from the aforementioned 164 individuals was mapped to this combined reference using Bowtie 2 [27] in its --very-sensitive-local mode. After this, Picard was used to mark and remove duplicate reads. The remaining reads were then filtered using Samtools to exclude reads that were low quality, non-primary, or supplementary (samtools view -h -q 10 -F 0x904). The final list of read counts was processed using a custom Python script to create an array of reads per feature per individual. TE copy numbers were estimated by first removing the reads mapping to mitochondrial, chloroplast, and rRNA genes. Due to the high quality assembly and annotation of the genome, the remaining genes were assumed to exist in single copy. The average coverage per feature *j* (i.e., gene or transposon) in individual *i* was computed as *c*_*ij*_ = *r*_*ij*_*l*_*ij*_/*k*_*j*_, where *r*_*ij*_ and *l*_*ij*_ are the number and length of reads to feature *j* in individual *i*, and *k*_*j*_ is the annotated length of feature *j* in the reference genome. To control for genes that might be present in more than single copy, the top 99th percentile of genes were removed. Writing *G* for the index set of the *N* = 33, 233 remaining genes, the average coverage was computed as $g_{i}=\frac{1}{N}\sum_{j\in G}c_{ij}$. The total copy number of TE features in each individual was estimated as ${\hat{c}}_{ij}=c_{ij}/g_{i}$. Bowtie 2, Picard, and Samtools can be downloaded from:

- http://bowtie-bio.sourceforge.net/bowtie2/index.shtml
- https://broadinstitute.github.io/picard/
- http://www.htslib.org/

The second data set (analyzed in Fig 2) comes from an analysis of 131 lines of *Drosophila melanogaster* obtained from the Drosophila Genetic Reference Panel [16]. The individual lines were derived from a large population in Raleigh, North Carolina. In a previously published analysis, Cridland et al. used genomic DNA sequencing to identify over 17,000 TE insertions across these lines. For each insertion (locus), in each individual, this previous work provides a call of present, absent, or indeterminate. Because the vast majority of TE insertions were determined to be rare (83% are present in only one line), we treated loci with indeterminate calls as absent. Elements that were not previously identified as transposons (DNA intermediates) or retrotransposons (RNA intermediate) were excluded. Chromosome 4 was excluded from this analysis, because it is known to have a number of peculiar features (e.g., small size and lack of recombination) [28].

## Supporting information

S1 File Data and scripts. This compressed directory contains the two data sets discussed in ‘Appendix: Data Analysis’ (above) and the scripts used to generate Figs 1 and 2.
(ZIP)

S1 Text Derivations and model formulation. This supporting text derives the moment equations for TE load from the master equation model. The text also provides details of model formulation including moment closure techniques, how selection is incorporated into the master equation and moment-based models, and numerical methods.
(PDF)
