# Supplementary material for: Population genetics of transposable element load: A mechanistic account of observed overdispersion
Source: PLoS One. 2022 Jul 14;17(7):e0270839. doi: 10.1371/journal.pone.0270839 (PMC9282655; doi:10.1371/journal.pone.0270839)
Supplement: S1 Text — This supporting text derives the moment equations for TE load from the master equation model. The text also provides details of model formulation including moment closure techniques, how selection is incorporated into the master equation and moment-based models, and numerical methods. (PDF) [file pone.0270839.s002.pdf]

Supplementary Information

S1 Text

Population genetics of transposable element load: a mechanistic account of observed overdispersion

Ronald D. Smith[1], Joshua R. Puzey[2◕], Gregory D. Conradi Smith[1◕*],

1 Department of Applied Science, William & Mary, Williamsburg, VA, USA
2 Department of Biology, William & Mary, Williamsburg, VA, USA

◕ These authors contributed equally to this work.
*Corresponding author. Email: greg@wm.edu

1 Comments on the classical model

In classical TE population genetics [1], the state of an infinite diploid population at a given chromosomal site i, for $0 \leq i \leq m$, is described by its frequency, x_i, where $0 \leq x_i \leq 1$. Assuming insertion sites exhibit no linkage disequilibrium, the set of frequencies, $\{x_i\}_{i=1}^{m}$, describes the state of the population. The TE load of a randomly sampled diploid individual is a random number $\mathbf{n}$ given by

$$\mathbf{n} = \sum_{i=1}^{m} \mathbf{x}_i + \sum_{i=1}^{m} \mathbf{y}_i \,. \tag{A1}$$

where $\mathbf{x}_i$ and $\mathbf{y}_i$ are pairs of i.i.d. Bernoulli random variables with parameters x_i. Thus, $\mathsf{E}[\mathbf{x}_i] = \mathsf{E}[\mathbf{y}_i] = x_i$ and the mean copy number of TEs per individual is

$$\bar{n} = \mathsf{E}[\mathbf{n}] = \sum_{i=1}^{m} \mathsf{E}[\mathbf{x}_i] + \sum_{i=1}^{m} \mathsf{E}[\mathbf{y}_i] = 2\sum_{i=1}^{m} x_i = 2m\bar{x}$$

where in the last equality we have written $\bar{n}$ in terms of the number of loci and the mean frequency, $\bar{x} = (1/m)\sum_{i=1}^{m} x_i$. The variance of a Bernoulli random variable with parameter x_i is $x_i(1 - x_i)$. As a consequence, the variance of TE load is

$$\mathsf{Var}[\mathbf{n}] = \sum_{i=1}^{m} \mathsf{Var}[\mathbf{x}_i + \mathbf{y}_i] = 2\sum_{i=1}^{m} x_i(1 - x_i)\,.$$

The diploid load given by Eq (A1) is the sum of two i.i.d. Poisson binomial random variables, that is, $\mathbf{n} = \mathbf{X} + \mathbf{Y}$ where $\mathbf{X} = \sum_{i=1}^{m} \mathbf{x}_i$ is the sum of independent Bernoulli random variables that are not necessarily independent (and similarly for $\mathbf{Y} = \sum_{i=1}^{m} \mathbf{y}_i$). It is well-known that

$$\mathsf{Var}[\mathbf{X}] = m\bar{x}(1 - \bar{x}) - m\sigma_x^2\,. \tag{A2}$$

where $\sigma_x^2 = (1/m)\sum_{i=1}^m (x_i - \bar{x})^2$ is the "variance" among the parameters of the Poisson binomial distribution, $\{x_i\}_{i=1}^m$ (i.e., the variability of frequencies of occupation of the TE loci). Using $\mathsf{Var}[\mathbf{Y}] = \mathsf{Var}[\mathbf{X}]$, $\mathsf{Var}[\mathbf{n}] = 2\mathsf{Var}[\mathbf{X}]$, and Eq (A2), the variance of TE load is

$$\mathsf{Var}[\mathbf{n}] = 2m\bar{x}(1-\bar{x}) - 2m\sigma_x^2 \,.$$

Substituting $\bar{x} = \bar{n}/2m$ in the above expression gives

$$V_n \approx \bar{n}\left(1 - \frac{\bar{n}}{2m}\right) - 2m\sigma_x^2 \,,$$

which is Eq (6) in the main text.

To extend this model of TE population genetics to include the effect of natural selection, ref. [1] assumes a viability function, w_n, that is a decreasing function of total genome-wide TE load ($dw_n/dn < 0$). The effect of selection is to decrease the occupation frequency at each loci in a manner that is proportional to $\frac{1}{2}x_i(1-x_i)$, which is the variance of a Bernoulli random variable with parameter x_i, and also proportional the derivative, with respect to x_i, of the mean fitness of the population, $\bar{w} = \mathsf{E}[w_\mathbf{n}]$,

$$\Delta x_i = \frac{x_i(1-x_i)}{2\bar{w}}\frac{\partial \bar{w}}{\partial x_i} = x_i(1-x_i)\frac{\partial \ln \bar{w}}{\partial \bar{n}} \,.$$

The second equality is obtained using $(1/x_i)\partial\bar{w}/\partial x_i = \partial \ln \bar{w}/\partial x_i$ and noting that $\bar{n} = 2\sum_{i=1}^m x_i$ implies $\partial\bar{n}/\partial x_i = 2$. Summing over all sites gives

$$\Delta\bar{n} = 2\sum_{i=1}^{m} \Delta x_i = \bar{n}\left(1 - \frac{\bar{n}}{2m}\right)\frac{\partial \ln \bar{w}}{\partial \bar{n}} \,.$$

Using $V_n = \bar{n}(1 - \bar{n}/2m)$ and approximating the mean fitness of the population ($\bar{w} = \mathsf{E}[w_\mathbf{n}]$) by the fitness of an individual with an average number of copies ($w_{\bar{n}}$) gives the first term (boxed below) of Eq (3) in the main text,

$$\Delta\bar{n} \approx \boxed{V_n \frac{d \ln w_{\bar{n}}}{d\bar{n}}} + \bar{n}(u_{\bar{n}} - \nu) + \frac{V_n}{2}\left(2\frac{du_{\bar{n}}}{d\bar{n}} + \bar{n}\frac{d^2 u_{\bar{n}}}{d\bar{n}^2}\right) \,.$$

2 Statistics of indistinguishable TE families

For TE families with independent proliferation and excision dynamics, the dispersion of TE load that results when families are not distinguished is always *less* than the overdispersion of at least one of the TE families. To see this, consider the generalization of main text Eq (11) to an arbitrary number of TE families,

$$\mathsf{Var}[\mathbf{x}] = \mathsf{Var}[\textstyle\sum_i \mathbf{x}_i] = \sum_i \mathsf{Var}[\mathbf{x}_i] + 2\sum_{i<j}\mathsf{Cov}[\mathbf{x}_i, \mathbf{x}_j] = \sum_i \bar{n}_i F_i + 2\sum_{i<j}\mathsf{Cov}[\mathbf{x}_i, \mathbf{x}_j] \,.$$

Substituting $\mathsf{Var}[\mathbf{x}_i] = \bar{n}_i F_i$ and dividing by $\bar{n} = \sum_i \bar{n}_i$ gives an expression for the composite index of dispersion,

$$F = \frac{\mathsf{Var}[\mathbf{x}]}{\bar{n}} = \frac{\sum_i \bar{n}_i F_i}{\bar{n}} + \frac{2}{\bar{n}}\textstyle\sum_{i<j}\mathsf{Cov}[\mathbf{x}_i, \mathbf{x}_j] \,.$$

Assuming that the within-population loads for the families of TEs are independent, the covariances will be zero. In that case,

$$F = \textstyle\sum_i p_i F_i \,,$$

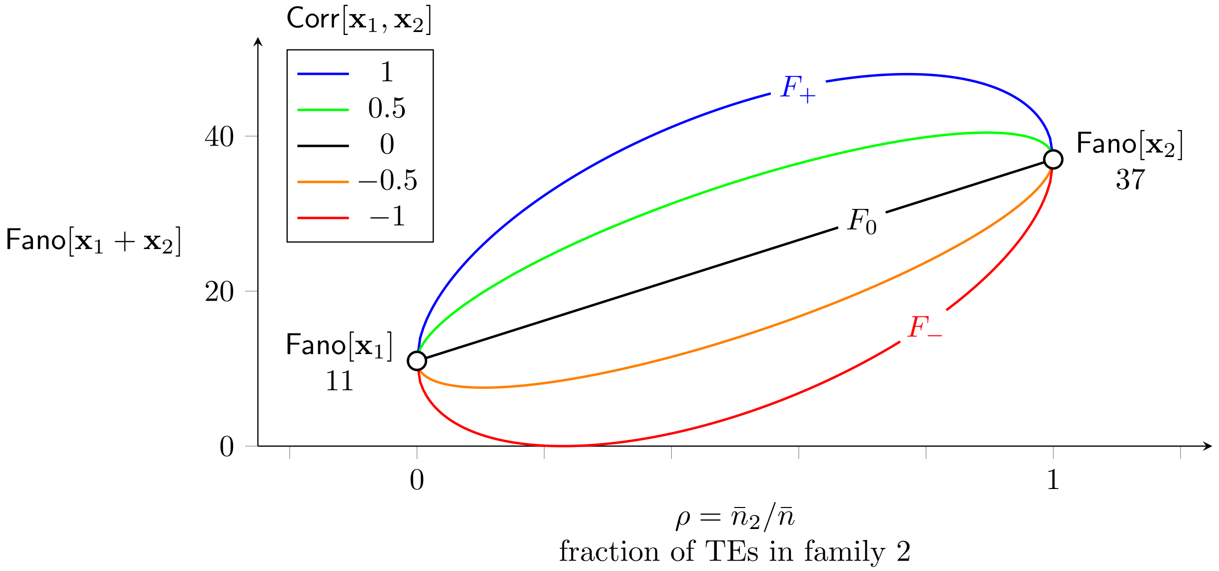

Fig A1. The composite index of dispersion for two TE families. The Fano factor $\mathsf{Fano}[\mathbf{x}_1 + \mathbf{x}_2]$ given by Eqs (A3–A4) is plotted as a function of the fraction of TEs in the second of two families ($\rho = \bar{n}_2/\bar{n}$). This composite index of dispersion depends on the Fano factors of both families, $\mathsf{Fano}[\mathbf{x}_1]$ and $\mathsf{Fano}[\mathbf{x}_2]$, and the correlation coefficient ($\mathsf{Corr}[\mathbf{x}_1, \mathbf{x}_2]$) for the loads of the families within the population.

where $p_i = \bar{n}_i/\bar{n}$. Thus, the composite Fano factor F is in the range, $\min_i F_i \le F \le \max_i F_i$.

The situation is more complicated when the dynamics of TE families are not independent. For simplicity, consider the case of two TE families. Beginning with Eq (11) of the main text,

$$\begin{aligned} \mathsf{Var}[\mathbf{x}] &= \mathsf{Var}[\mathbf{x}_1 + \mathbf{x}_2] = \mathsf{Var}[\mathbf{x}_1] + \mathsf{Var}[\mathbf{x}_2] + 2\mathsf{Cov}[\mathbf{x}_1, \mathbf{x}_2] \\ &= \bar{n}_1 F_1 + \bar{n}_2 F_2 + 2\mathsf{Cov}[\mathbf{x}_1, \mathbf{x}_2]\,, \end{aligned}$$

and the definition of the correlation of two random variables,

$$\mathsf{Corr}[\mathbf{x}_1, \mathbf{x}_2] = \frac{\mathsf{Cov}[\mathbf{x}_1, \mathbf{x}_2]}{\sqrt{\mathsf{Var}[\mathbf{x}_1]\mathsf{Var}[\mathbf{x}_2]}}\,,$$

the composite Fano factor is,

$$F = \frac{1}{\bar{n}_1 + \bar{n}_2}\left(\bar{n}_1 F_1 + \bar{n}_2 F_2 + 2\sqrt{\bar{n}_1 F_1\, \bar{n}_2 F_2}\,\mathsf{Corr}[\mathbf{x}_1, \mathbf{x}_2]\right) \tag{A3}$$

$$= (1-\rho)F_1 + \rho F_2 + 2\sqrt{(1-\rho)F_1\, \rho F_2}\,\mathsf{Corr}[\mathbf{x}_1, \mathbf{x}_2]\,. \tag{A4}$$

In this expression, $\rho = \bar{n}_2/(\bar{n}_1 + \bar{n}_2)$, the fraction of TEs in the second family, takes values between 0 and 1, and the correlation function, $\mathsf{Corr}[\mathbf{x}_1, \mathbf{x}_2]$, takes values between -1 and 1. When there is no correlation ($\mathsf{Corr}[\mathbf{x}_1, \mathbf{x}_2] = 0$), the composite Fano factor is a weighted average of F_1 and F_2, namely,

$$F_0 = (1-\rho)F_1 + \rho F_2\,. \tag{A5}$$

For fixed ρ, F is maximized when $\mathsf{Corr}[\mathbf{x}_1, \mathbf{x}_2] = 1$,

$$F_+ = \left[\sqrt{(1-\rho)F_1} + \sqrt{\rho F_2}\right]^2 > F_0\,.$$

Similarly, F is minimized when $\mathsf{Corr}[\mathbf{x}_1, \mathbf{x}_2] = -1$,

$$F_- = \left[\sqrt{(1-\rho)F_1} - \sqrt{\rho F_2}\right]^2 < F_0 \, .$$

It can be shown that $\max_\rho F_+ = F_1 + F_2$ and $\min_\rho F_- = 0$. For less extreme values of the correlation of TE family loads, we have

$$\mathsf{Corr}[\mathbf{x}_1, \mathbf{x}_2] < 0 \implies F < F_0$$
$$\mathsf{Corr}[\mathbf{x}_1, \mathbf{x}_2] > 0 \implies F > F_0$$

for all $0 \leq \rho \leq 1$ (see Fig A1). Notably, the composite Fano factor can either increase or decrease when families of TEs are lumped into larger groups, or split into smaller groups. The composite Fano factor will be greater or less than the weighted average given by Eq (A5) depending on whether the TE loads are positively or negatively correlated in the population.

For example, the overdispersion documented in Fig 1 of the main text can be analyzed at the level of family, class, or *in toto*. At the level of class, Table 1 of the main text shows Fano factors for *M. guttatus* of 61 for Class I and 646 for Class II. When the two classes of TEs are combined, the overall Fano factor is 525, which is an intermediate value ($61 < 525 < 646$). This composite Fano factor is slightly higher than would be obtained under the assumption of independence, which is $(1-\rho)\,61 + \rho\,646 = 513$, as in Eq (A5), where we have used the proportion of TEs in each class ($\rho = 0.772$). This calculation is consistent with the small positive correlation between the TE loads for the two classes ($\mathsf{Corr}[\mathbf{x}_{\text{Class I}}, \mathbf{x}_{\text{Class II}}] = 0.0734$).

When analyzed at the level of family, the Fano factors for Class I elements of *M. guttatus* range from 7 to 31, while for Class II elements the range is 12 to 228 (see Fig 1 of main text). These ranges are less than the indices of dispersion observed at the level of class (61 and 646, respectively), because the loads for TE families (within each class) are positively correlated. The symmetric matrix of correlation coefficients at the level of family are shown in Table A1. Note that the small positive and negative correlations are predominantly located within the off-diagonal blocks, where loads of Class I and II elements are being compared.

Table A1. Correlation of TE load at the level of family in *Mimulus guttatus*.

	CACTA	Helitron	MULE	PIF-H	Tc1-M	hAT	Copia	Gypsy	L1	SINE
CACTA	1.0	0.79	0.84	0.77	0.79	0.88	0.39	0.14	0.18	-0.48
Helitron	0.79	1.0	0.83	0.79	0.74	0.87	0.19	0.14	0.21	-0.3
MULE	0.84	0.83	1.0	0.92	0.95	0.92	0.17	0.032	-0.059	-0.24
PIF-Harbinger	0.77	0.79	0.92	1.0	0.92	0.89	0.064	-0.039	-0.011	-0.2
Tc1-Mariner	0.79	0.74	0.95	0.92	1.0	0.91	0.065	-0.13	-0.13	-0.25
hAT	0.88	0.87	0.92	0.89	0.91	1.0	0.15	-0.048	0.054	-0.41
Copia	0.39	0.19	0.17	0.064	0.065	0.15	1.0	0.84	0.49	0.012
Gypsy	0.14	0.14	0.032	-0.039	-0.13	-0.048	0.84	1.0	0.53	0.23
L1	0.18	0.21	-0.059	-0.011	-0.13	0.054	0.49	0.53	1.0	0.11
SINE	-0.48	-0.3	-0.24	-0.2	-0.25	-0.41	0.012	0.23	0.11	1.0

The six Class II elements (DNA transposons) are listed first, followed by four Class I elements (retrotransposons). The on- and off-diagonal blocks of this matrix correspond to comparisons of TE families within and between classes.

3 Derivation of moment equations with gain and loss terms

Let $\mathbf{n}$ be a random variable for the TE load of a randomly sampled diploid individual, and $\mathbf{x}$ and $\mathbf{y}$ be random variables for the TE load of randomly sampled haploid genomes (gametes). Define the moments of the probability distribution of haploid TE loads (given by p_i for $0 \le i \le m$) as

$$\mu_q = \mathsf{E}[\mathbf{x}^q] = \sum_{n=0}^{m} n^q p_n \quad \text{for} \quad q = 0, 1, 2, \cdots \tag{A6}$$

where $\mu_0 = 1$ (conservation of probability), $\mu_1 = \mathsf{E}[\mathbf{x}]$ and $\mathsf{Var}[\mathbf{x}] = \mathsf{E}[\mathbf{x}^2] - \mathsf{E}[\mathbf{x}]^2 = \mu_2 - \mu_1^2$. Assuming random mating for which the diploid TE load is the sum of two i.i.d. gametic loads ($\mathbf{n} = \mathbf{x} + \mathbf{y}$), the mean and variance of the within-population diploid TE load are related to μ_2 and μ_1 as follows,

$$\mathsf{E}[\mathbf{n}] = 2\mathsf{E}[\mathbf{x}] = 2\mu_1 \tag{A7}$$

$$\mathsf{Var}[\mathbf{n}] = 2\mathsf{Var}[\mathbf{x}] = 2(\mu_2 - \mu_1^2)\,. \tag{A8}$$

Using Eqs (A7–A8) we see that the Fano factor of the diploid load is

$$\mathsf{Fano}[\mathbf{n}] = \frac{\mathsf{Var}[\mathbf{n}]}{\mathsf{E}[\mathbf{n}]} = \frac{2(\mu_2 - \mu_1^2)}{2\mu_1} = \frac{\mu_2}{\mu_1} - \mu_1\,.$$

Because the factor of two occurs in both the numerator and denominator of the above expression, the dispersion of the diploid load is equal to that of the haploid load, i.e. $\mathsf{Fano}[\mathbf{n}] = \mathsf{Fano}[\mathbf{x}]$.

3.1 ODEs for the dynamics of μ_1 and μ_2

The moment equations for the haploid TE load are derived by differentiating Eq (A6) to obtain

$$\frac{d\mu_q}{dt} = \sum_{n=0}^{m} n^q \frac{dp_n}{dt}\,.$$

Substituting Eqs (13–15) of the main text into the above expression gives

$$\frac{d\mu_q}{dt} = \sum_{n=0}^{m} n^q [g_{n-1}p_{n-1} - (g_n + \ell_n)p_n + \ell_{n+1}p_{n+1}] \tag{A9}$$

where it is understood that $g_{-1}p_{-1}$ (for $n = 0$) and $\ell_{m+1}p_{m+1}$ (for $n = m$) are not included in the sum, because these terms are absent in Eqs (13) and (15), respectively. Alternatively, for $q \ge 1$ Eq (A9) may be written as follows,

$$\frac{d\mu_q}{dt} = \sum_{n=1}^{m-1} n^q [g_{n-1}p_{n-1} - (g_n + \ell_n)p_n + \ell_{n+1}p_{n+1}] + m^q [g_{m-1}p_{m-1} - (g_m + \ell_m)p_m]\,,$$

because the $n = 0$ terms do not contribute. Rearranging this expression gives

$$\frac{d\mu_q}{dt} = \underbrace{-\sum_{n=0}^{m} n^q \ell_n p_n + \sum_{n=0}^{m-1} n^q \ell_{n+1} p_{n+1}}_{L_q} \underbrace{-\sum_{n=0}^{m} n^q g_n p_n + \sum_{n=1}^{m} n^q g_{n-1} p_{n-1}}_{G_q}\,.$$

The terms L_q are evaluated as follows,

$$\begin{aligned} L_q &= \sum_{n=0}^{m-1} n^q \ell_{n+1} p_{n+1} - \sum_{n=0}^{m} n^q \ell_n p_n = \sum_{n=1}^{m} (n-1)^q \ell_n p_n - \sum_{n=1}^{m} n^q \ell_n p_n \\ &= \sum_{n=1}^{m} [(n-1)^q - n^q] \ell_n p_n \,. \end{aligned}$$

For $q = 1$, $[(n-1)^q - n^q] = -1$; thus, L_1 is given by

$$L_1 = -\sum_{n=1}^{m} \ell_n p_n = -\sum_{n=0}^{m} \nu n p_n = -\nu \sum_{n=0}^{m} n p_n = -\nu \mu_1 \,, \tag{A10}$$

where in the second equality we use $\ell_n = \nu n$ and $\ell_0 = 0$. Similarly,

$$\begin{aligned} G_q &= \sum_{n=1}^{m} n^q g_{n-1} p_{n-1} - \sum_{n=0}^{m} n^q g_n p_n = \sum_{n=0}^{m-1} (n+1)^q g_n p_n - \sum_{n=0}^{m} n^q g_n p_n \\ &= \sum_{n=0}^{m-1} [(n+1)^q - n^q] g_n p_n - m^q g_m p_m \,. \end{aligned}$$

For $q = 1$, $[(n+1)^q - n^q] = +1$; thus, G_1 is given by

$$\begin{aligned} G_1 &= \sum_{n=0}^{m-1} g_n p_n - m g_m p_m = \sum_{n=0}^{m-1} (\eta_0 + \eta n)(1 - n/m) p_n - m(\eta_0 + \eta m) \underbrace{(1 - m/m)}_{0} p_m \\ &= \sum_{n=0}^{m} (\eta_0 + \eta n)(1 - n/m) p_n \,, \end{aligned} \tag{A11}$$

where we use $g_n = (\eta_0 + \eta n)(1 - n/m)$ and $g_m = 0$. Continuing the calculation, we find

$$\begin{aligned} G_1 &= \eta_0 \sum_{n=0}^{m} (1 - n/m) p_n + \eta \sum_{n=0}^{m} n(1 - n/m) p_n \\ &= \eta_0 \sum_{n=0}^{m} p_n - (\eta_0/m) \sum_{n=0}^{m} n p_n + \eta \sum_{n=0}^{m} n p_n + (\eta/m) \sum_{n=0}^{m} n^2 p_n \,. \end{aligned}$$

This derivation shows that G_1 is given by

$$G_1 = \eta_0 - \eta_0 \mu_1/m + \eta \mu_1 - \eta \mu_2/m \,. \tag{A12}$$

Combining the expressions for L_1 and G_1 gives the ODE for μ_1, the first moment of haploid TE load,

$$\frac{d\mu_1}{dt} = \eta_0[1 - \mu_1/m] - \nu \mu_1 + \eta[\mu_1 - \mu_2/m] = L_1 + G_1 \,. \tag{A13}$$

Similar calculations give $d\mu_0/dt = 0$ (conservation of probability) and

$$L_2 = \nu \mu_1 - 2\nu \mu_2 \tag{A14}$$

$$G_2 = \eta_0[1 - \mu_1/m] + 2\eta_0[\mu_1 - \mu_2/m] + \eta[\mu_1 - \mu_2/m] + 2\eta[\mu_2 - \mu_3/m] \,. \tag{A15}$$

Using these expressions, the ODE for the second moment, $d\mu_2/dt = L_2 + G_2$, is found to be

$$\frac{d\mu_2}{dt} = \eta_0[1 - \mu_1/m] + 2\eta_0[\mu_1 - \mu_2/m] + \nu\mu_1 - 2\nu\mu_2 + \eta[\mu_1 - \mu_2/m] + 2\eta[\mu_2 - \mu_3/m] \,. \quad \text{(A16)}$$

Eqs (A13–A16) are the first two ODEs in a sequence for which $d\mu_1/dt$ depends on μ_1 and μ_2, $d\mu_2/dt$ depends on μ_1, μ_2 and μ_3, and so on, as follows,

$$\frac{d\mu_1}{dt} = f_1(\mu_1, \mu_2) \quad \text{(A17)}$$

$$\frac{d\mu_2}{dt} = f_2(\mu_1, \mu_2, \mu_3) \quad \text{(A18)}$$

$$\vdots$$

$$\frac{d\mu_q}{dt} = f_q(\mu_{q-1}, \mu_q, \mu_{q+1}) \,. \quad \text{(A19)}$$

Sec 5 shows how this open system of ODEs can be closed by assuming an algebraic relationship between the third moment and those of lower order. The influence of selection on the both the master equation and moment equation models is discussed in Sec 4. The following two sections explore parameter regimes for which the moment equations decouple and it is possible to derive analytical steady states for the population mean and variance of TE load.

3.2 Moment ODEs in absence of copy-and-paste transposition

When there is no copy-and-paste transposition ($\eta = 0$), Eqs (A13–A16) simplify as follows:

$$\begin{aligned} \frac{d\mu_1}{dt} &= \eta_0(1 - \mu_1/m) - \nu\mu_1 \\ \frac{d\mu_2}{dt} &= \eta_0(1 - \mu_1/m) + 2\eta_0(\mu_1 - \mu_2/m) + \nu\mu_1 - 2\nu\mu_2 \,. \end{aligned}$$

Notice that the dependence of $d\mu_1/dt$ on μ_2, and $d\mu_2/dt$ on μ_3, vanishes when $\eta = 0$. Regrouping terms gives

$$\begin{aligned} \frac{d\mu_1}{dt} &= \eta_0 - (\nu + \eta_0/m)\mu_1 \\ \frac{d\mu_2}{dt} &= \eta_0 + (v + 2\eta_0 - \eta_0/m)\mu_1 - 2(\nu + \eta_0/m)\mu_2 \,. \end{aligned}$$

This system has steady state given by

$$\begin{aligned} \mu_1 &= \frac{\eta_0}{\nu + \eta_0/m} = \frac{m\,\eta_0/\nu}{m + \eta_0/\nu} \\ \mu_2 &= \frac{\eta_0 + (\nu + 2\eta_0 - \eta_0/m)\mu_1}{2(\nu + \eta_0/m)} = \mu_1^2 + \frac{\nu}{\nu + \eta_0/m}\mu_1 \,. \end{aligned}$$

The central moment $\hat{\mu}_2 = \mu_2 - \mu_1^2$, the variance in haploid load, is thus

$$\hat{\mu}_2 = \frac{\nu}{\nu + \eta_0/m}\mu_1 = \frac{\eta_0\nu}{(\nu + \eta_0/m)^2} = \frac{m^2\,\eta_0/\nu}{(m + \eta_0/\nu)^2} \,.$$

Noting that $\mathsf{Var}[\mathbf{n}] = \sigma_n^2 = 2\hat{\mu}_2$ and $\mathsf{E}[\mathbf{n}] = \bar{n} = 2\mu_1$, we find

$$\begin{aligned}\bar{n} &= \frac{2\eta_0}{\nu + \eta_0/m} = \frac{2m\,\eta_0/\nu}{m + \eta_0/\nu} \\ \sigma_n^2 &= \frac{2\eta_0\nu}{(\nu + \eta_0/m)^2} = \frac{2m^2\,\eta_0/\nu}{(m + \eta_0/\nu)^2} = \frac{\nu\,\bar{n}}{\nu + \eta_0/m} = \frac{m\,\bar{n}}{m + \eta_0/\nu}\,.\end{aligned}$$

The index of dispersion for the diploid load is thus

$$\mathsf{Fano}[\mathbf{n}] = \frac{\nu}{\nu + \eta_0/m} = \frac{m}{m + \eta_0/\nu}\,.$$

3.3 Moment ODEs when occupiable loci are not limiting

When occupiable loci are not limiting ($\mu_1 << m$), we may consider Eqs (A13–A16) in the limit as $m \to \infty$,

$$\begin{aligned}\frac{d\mu_1}{dt} &= \eta_0 - \nu\mu_1 + \eta\mu_1 \\ \frac{d\mu_2}{dt} &= \eta_0 + 2\eta_0\mu_1 + \nu\mu_1 - 2\nu\mu_2 + \eta\mu_1 + 2\eta\mu_2\,.\end{aligned}$$

Note that the large m limit uncouples the moment ODEs. Regrouping terms,

$$\begin{aligned}\frac{d\mu_1}{dt} &= \eta_0 - (\nu - \eta)\mu_1 \\ \frac{d\mu_2}{dt} &= \eta_0 + (2\eta_0 + \eta + \nu)\mu_1 - 2(\nu - \eta)\mu_2\,.\end{aligned}$$

Provided $\nu > \eta$, this system has a stable steady state given by

$$\begin{aligned}\mu_1 &= \frac{\eta_0}{\nu - \eta} \\ \mu_2 &= \frac{\eta_0 + (2\eta_0 + \eta + \nu)\mu_1}{2(\nu - \eta)} = \mu_1^2 + \frac{\nu}{\nu - \eta}\mu_1\,.\end{aligned}$$

The central moment $\hat{\mu}_2 = \mu_2 - \mu_1^2$, the variance in haploid load, is thus

$$\hat{\mu}_2 = \frac{\nu}{\nu - \eta}\mu_1 = \frac{\eta_0\nu}{(\nu - \eta)^2}\,.$$

Noting that $\mathsf{Var}[\mathbf{n}] = \sigma_n^2 = 2\hat{\mu}_2$ and $\mathsf{E}[\mathbf{n}] = \bar{n} = 2\mu_1$ gives Eqs (27–28) of the main text, namely,

$$\begin{aligned}\bar{n} &= \frac{2\eta_0}{\nu - \eta} \\ \sigma_n^2 &= \frac{2\eta_0\nu}{(\nu - \eta)^2} = \frac{\nu\bar{n}}{\nu - \eta}\,.\end{aligned}$$

The index of dispersion for the diploid load is thus

$$\mathsf{Fano}[\mathbf{n}] = \frac{\nu}{\nu - \eta}\,,$$

as in Eq (29) of the main text.

3.4 Central moment equations

Recall that the open system of moment equations for the probability distribution of TE load takes the form

$$\begin{aligned}\frac{d\mu_1}{dt} &= \eta_0(1-\mu_1/m) - \nu\mu_1 + \eta(\mu_1 - \mu_2/m)\\ \frac{d\mu_2}{dt} &= \eta_0(1-\mu_1/m) + 2\eta_0(\mu_1-\mu_2/m) + \nu\mu_1 - 2\nu\mu_2 + \eta(\mu_1-\mu_2/m) + 2\eta(\mu_2-\mu_3/m)\,,\end{aligned}$$

where $d\mu_3/dt = f_3(\mu_2, \mu_3, \mu_4)$, and so on, as in Eqs (A17–A19). Rearranging terms in the equations for the first two moments gives

$$\begin{aligned}\frac{d\mu_1}{dt} &= \eta_0 - \left(\nu - \eta + \frac{\eta_0}{m}\right)\mu_1 - \frac{\eta}{m}\mu_2 && \text{(A20)}\\ \frac{d\mu_2}{dt} &= \eta_0 + \left(\nu + \eta + 2\eta_0 - \frac{\eta_0}{m}\right)\mu_1 - 2\left(\nu - \eta + \frac{\eta_0 + \eta/2}{m}\right)\mu_2 - \frac{2\eta}{m}\mu_3\,. && \text{(A21)}\end{aligned}$$

It is convenient to express Eqs (A20–A21) in terms of the central moments. The first central moment of the haploid TE load is the mean, $\mu_1 = \mathsf{E}[\mathbf{x}]$. The second central moment is the variance

$$\hat{\mu}_2 = \mathsf{Var}[\mathbf{x}] = \mathsf{E}[(\mathbf{x} - \mathsf{E}[\mathbf{x}])^2] = \mathsf{E}[\mathbf{x}^2] - \mathsf{E}[\mathbf{x}]^2 = \mu_2 - \mu_1^2\,.$$

The third central moment is

$$\hat{\mu}_3 = \mathsf{E}[(\mathbf{x} - \mathsf{E}[x])^3] = \mu_3 - 3\mu_1\mu_2 + 2\mu_1^3 = \mu_3 - 3\mu_1\hat{\mu}_2 - \mu_1^3\,. \qquad \text{(A22)}$$

To find an ODE for the dynamics of the variance, we differentiate $\hat{\mu}_2 = \mu_2 - \mu_1^2$ to obtain

$$\frac{d\hat{\mu}_2}{dt} = \frac{d\mu_2}{dt} - 2\mu_1\frac{d\mu_1}{dt}\,. \qquad \text{(A23)}$$

Substituting Eqs (A20–A21) into this expression we obtain,

$$\begin{aligned}\frac{d\mu_1}{dt} &= \eta_0 - \left(\nu - \eta + \frac{\eta_0}{m}\right)\mu_1 - \frac{\eta}{m}\left(\hat{\mu}_2 + \mu_1^2\right) && \text{(A24)}\\ \frac{d\hat{\mu}_2}{dt} &= \eta_0 + \left(\nu + \eta - \frac{\eta_0}{m}\right)\mu_1 - 2\left(\nu - \eta + \frac{\eta_0 + \eta/2}{m}\right)\hat{\mu}_2 - \frac{\eta}{m}(4\mu_1\hat{\mu}_2 + \mu_1^2 + 2\hat{\mu}_3)\,. && \text{(A25)}\end{aligned}$$

Using $\mathsf{E}[\mathbf{n}] = \bar{n} = 2\mu_1$ and $\mathsf{Var}[\mathbf{n}] = \sigma_n^2 = 2\hat{\mu}_2$, and $\mathsf{E}[(\mathbf{n} - \bar{n})^3] = 2\hat{\mu}_3$, Eqs (A24–A25) may be transformed into equations for the mean and variance of diploid load. To see this, write $\mu_1 = \bar{n}/2$ and $\hat{\mu}_2 = \sigma_n^2/2$ and differentiate to obtain

$$\frac{1}{2}\frac{d\bar{n}}{dt} = \frac{d\mu_1}{dt} \quad \text{and} \quad \frac{1}{2}\frac{d\sigma_n^2}{dt} = \frac{d\hat{\mu}_2}{dt}\,.$$

Substitution gives

$$\begin{aligned}\frac{1}{2}\frac{d\bar{n}}{dt} &= \eta_0 - \frac{1}{2}\left(\nu - \eta + \frac{\eta_0}{m}\right)\bar{n} - \frac{\eta}{2m}\left(\sigma_n^2 + \frac{\bar{n}^2}{2}\right)\\ \frac{1}{2}\frac{d\sigma_n^2}{dt} &= \eta_0 + \frac{1}{2}\left(\nu + \eta - \frac{\eta_0}{m}\right)\bar{n} - \left(\nu - \eta + \frac{\eta_0 + \eta/2}{m}\right)\sigma_n^2 - \frac{\eta}{m}\left(\bar{n}\sigma_n^2 + \frac{\bar{n}^2}{4} + \mathsf{E}[(\mathbf{n} - \bar{n})^3]\right)\,,\end{aligned}$$

where we gave used Eq (A22). After simplifying, these equations become

$$\frac{d\bar{n}}{dt} = 2\eta_0 - \left(\nu - \eta + \frac{\eta_0}{m}\right)\bar{n} - \frac{\eta}{m}\left(\sigma_n^2 + \frac{\bar{n}^2}{2}\right) \tag{A26}$$

$$\begin{aligned}\frac{d\sigma_n^2}{dt} &= 2\eta_0 + \left(\nu + \eta - \frac{\eta_0}{m}\right)\bar{n} - 2\left(\nu - \eta + \frac{\eta_0 + \eta/2}{m}\right)\sigma_n^2 \\ &- \frac{2\eta}{m}\left(\bar{n}\sigma_n^2 + \frac{\bar{n}^2}{4} + \mathsf{E}[(\mathbf{n} - \bar{n})^3]\right) .\end{aligned} \tag{A27}$$

Taking the limit as $m \to \infty$ gives

$$\begin{aligned}\frac{d\bar{n}}{dt} &= 2\eta_0 - (\nu - \eta)\bar{n} \\ \frac{d\sigma_n^2}{dt} &= 2\eta_0 + (\nu + \eta)\bar{n} - 2(\nu - \eta)\sigma_n^2 ,\end{aligned}$$

which are Eqs (25–26) of the main text.

4 Selection in the master equation and moment equation models

In the master equation formulation, p_n is the probability of randomly sampling a gamete with a TE load of n. Under the assumption of random mating, selection leads to the following probabilities for each load in the next generation,

$$p_i' = \frac{p_i \sum_j w_{i+j} p_j}{\sum_i p_i \sum_j w_{i+j} p_j} = \frac{p_i \bar{w}_i}{\sum_i p_i \bar{w}_i} = \frac{p_i \bar{w}_i}{\bar{w}} \qquad 0 \le i, j \le m$$

where

$$\bar{w} = \mathsf{E}[w_{\mathbf{n}}] = \sum_i p_i \sum_j p_j w_{i+j} = \sum_{i,j} p_i p_j w_{i+j}$$

is the mean fitness of the diploid population. Selection may be included in the master equations for TE load, Eqs (20–22), in the following manner:

$$\frac{dp_n}{dt} = \alpha(p_n' - p_n) + \cdots = \alpha\left(\frac{p_n \bar{w}_n}{\bar{w}} - p_n\right) + \cdots = \alpha\frac{p_n(\bar{w}_n - \bar{w})}{\bar{w}} + \cdots$$

where for typographical convenience we do not write the reaction terms involving u_n and v_n (these are indicated by $\cdots$). In the weak selection limit, $w_n = (1-s)^n \approx 1 - sn$ and the mean fitness $\bar{w}$ becomes

$$\bar{w} = \sum_n p_n \bar{w}_n \approx \sum_n p_n[1 - sn - s\mu_1] = 1 - s\mu_1 - s\sum_n np_n = 1 - 2s\mu_1 .$$

Thus, weak selection can be included in the moment equations as follows

$$\frac{dp_n}{dt} = \alpha p_n \frac{1 - sn - s\mu_1 - (1 - 2s\mu_1)}{1 - 2s\mu_1} + \cdots = \frac{\alpha s}{1 - 2s\mu_1} p_n(\mu_1 - n) + \cdots .$$

This expression leads to the following differential equation for the first moment in the weak selection limit,

$$\frac{d\mu_1}{dt} = \sum_n n\frac{dp_n}{dt} = \frac{\alpha s}{1 - 2s\mu_1}\left(\mu_1 \sum_n np_n - \sum_n n^2 p_n\right) + L_1 + G_1 \tag{A28}$$

$$= -\frac{\alpha s}{1 - 2s\mu_1}\left[\mu_2 - \mu_1^2\right] + L_1 + G_1 , \tag{A29}$$

where the quantity in brackets is the variance in haploid load ($\hat{\mu}_2 = \mu_2 - \mu_1^2$) and V_1 and G_1 are given by Eqs (A10–A12). A similar calculation gives the dynamics of the second moment of the haploid load,

$$\frac{d\mu_2}{dt} = \sum_n n^2 \frac{dp_n}{dt} = \frac{\alpha s}{1-2s\mu_1}\left(\mu_1 \sum_n n^2 p_n - \sum_n n^3 p_n\right) + L_2 + G_2 \quad \text{(A30)}$$

$$= -\frac{\alpha s}{1-2s\mu_1}\left[\mu_3 - \mu_1\mu_2\right] + L_2 + G_2\,. \quad \text{(A31)}$$

where L_2 and G_2 are given by Eqs (A14–A15). Using Eq (A23), we may derive the following ODE for the variance in haploid load,

$$\begin{aligned}\frac{d\hat{\mu}_2}{dt} &= \frac{d\mu_2}{dt} - 2\mu_1\frac{d\mu_1}{dt} \\ &= -\frac{\alpha s}{1-2s\mu_1}\left[(\mu_3 - \mu_1\mu_2) - 2\mu_1(\mu_2 - \mu_1^2)\right] + \cdots \\ &= -\frac{\alpha s}{1-2s\mu_1}\left[\mu_3 - 3\mu_1\mu_2 + 2\mu_1^3\right] + \cdots \\ &= -\frac{\alpha s}{1-2s\mu_1}\hat{\mu}_3 + \cdots\,.\end{aligned}$$

Using $\bar{n} = 2\mu_1$ and $d\bar{n}/dt = 2d\mu_1/dt$, and $\sigma_n^2/2 = \mu_2 - \mu_1^2$, we see that Eq (A29) is equivalent to

$$\frac{d\bar{n}}{dt} = -\frac{\alpha s}{1-s\bar{n}}\sigma_n^2 + \cdots\,.$$

Using $\mathsf{E}[(\mathbf{n}-\bar{n})^3] = 2\hat{\mu}_3$ and $\sigma_n^2/2 = \hat{\mu}_2$, we obtain

$$\frac{d\sigma_n^2}{dt} = -\frac{\alpha s}{1-s\bar{n}}\mathsf{E}[(\mathbf{n}-\bar{n})^3] + \cdots\,.$$

Combining these results for the effect of selection with the reaction terms of the neutral model, given by Eqs (A26–A27), we obtain the following equations for the mean and variance of diploid load under the influence of selection:

$$\frac{d\bar{n}}{dt} = -\frac{\alpha s}{1-s\bar{n}}\cdot\sigma_n^2 + 2\eta_0 - \left(\nu - \eta + \frac{\eta_0}{m}\right)\bar{n} - \frac{\eta}{m}\left(\sigma_n^2 + \frac{\bar{n}^2}{2}\right) \quad \text{(A32)}$$

$$\begin{aligned}\frac{d\sigma_n^2}{dt} &= -\frac{\alpha s}{1-s\bar{n}}\cdot\mathsf{E}[(\mathbf{n}-\bar{n})^3] + 2\eta_0 + \left(\nu + \eta - \frac{\eta_0}{m}\right)\bar{n} - 2\left(\nu - \eta + \frac{\eta_0 + \eta/2}{m}\right)\sigma_n^2 \\ &- \frac{2\eta}{m}\left(\bar{n}\sigma_n^2 + \frac{\bar{n}^2}{4} + \mathsf{E}[(\mathbf{n}-\bar{n})^3]\right)\,. \quad \text{(A33)}\end{aligned}$$

Taking the limit of Eqs (A32–A33) as $m \to \infty$ gives

$$\begin{aligned}\frac{d\bar{n}}{dt} &= -\frac{\alpha s}{1-s\bar{n}}\cdot\sigma_n^2 + 2\eta_0 - (\nu-\eta)\bar{n} \\ \frac{d\sigma_n^2}{dt} &= -\frac{\alpha s}{1-s\bar{n}}\cdot\mathsf{E}[(\mathbf{n}-\bar{n})^3] + 2\eta_0 + (\nu+\eta)\bar{n} - 2(\nu-\eta)\sigma_n^2\,,\end{aligned}$$

which are Eqs (35–36) of the main text.

5 Moment closure

To analyze solutions of Eqs (A32–A33) without assuming that m is large or η is zero, the dependence of $d\sigma_n^2/dt$ on $\mathsf{E}[(\mathbf{n}-\bar{n})^3]$ (equivalently, the dependence of $d\hat{\mu}_2/dt$ on $\hat{\mu}_3$) must be accounted for. This is accomplished using the technique of moment closure, whereby we assume an algebraic relationship of the form $\mu_3 = \psi(\mu_2, \mu_1)$ or

$$\hat{\mu}_3 = \psi(\hat{\mu}_2, \mu_1)\,. \tag{A34}$$

One way to motivate a particular choice of algebraic relationship ψ is to select a distribution with properties similar to those exhibited by the master equation simulations. Next, one derives the relation, Eq (A34), that would be exact if the model truly exhibited the selected distribution.

5.1 Negative binomial closure

One possibility we have investigated is the negative binomial distribution. This choice is motivated by a few key properties. First, the negative binomial distribution is supported on the non-negative integers. The probability mass function for a negative binomial random variable, $\mathbf{X} \sim \mathrm{NB}(r,p)$ for $r > 0$ and $p \in [0,1]$, is

$$\mathsf{P}[\mathbf{X}=k] = \binom{k+r-1}{k} p^r (1-p)^k\,, \quad k \in \{0,1,2,3,\ldots\}\,.$$

Second, overdispersion is a propery of the the negative binomial distribution. The mean $\mu_1 = E[\mathbf{X}]$, variance $\hat{\mu}_2 = \mu_2 - \mu_1^2$, and index of dispersion $\mathsf{Fano}[\mathbf{X}] = \hat{\mu}_2/\mu_1$ are given by

$$\mu_1 = \frac{r(1-p)}{p}\,, \quad \hat{\mu}_2 = \frac{r(1-p)}{p^2}\,, \quad \frac{\hat{\mu}_2}{\mu_1} = \frac{1}{p} \geq 1\,.$$

The third central moment is

$$\hat{\mu}_3 = \frac{r(2-3p+p^2)}{p^3} = \frac{r(p-1)(p-2)}{p^3}\,. \tag{A35}$$

Inverting the above expressions to give

$$p = \frac{\mu_1}{\hat{\mu}_2} \qquad r = \frac{\mu_1^2}{\hat{\mu}_2 - \mu_1}\,.$$

Substituting into Eq (A35) gives

$$\hat{\mu}_3 = \frac{2(\hat{\mu}_2)^2}{\mu_1} - \hat{\mu}_2 = \hat{\mu}_2\left(\frac{2\hat{\mu}_2 - \mu_1}{\mu_1}\right) =: \psi_{NB}(\hat{\mu}_2, \mu_1)\,. \tag{A36}$$

The corresponding expression for the third central moment of the diploid load is

$$\mathsf{E}[(\mathbf{n}-\bar{n})^3] = \sigma_n^2\left(\frac{2\sigma_n^2 - \bar{n}}{\bar{n}}\right)\,. \tag{A37}$$

5.2 Negative binomial closure: analysis of the weak selection limit

When Eqs (35–36) of the main text are modified consistent with the negative binomial moment closure (Sec 5.1), we obtain the closed system,

$$
\begin{aligned}
\frac{d\bar{n}}{dt} &= -\frac{\alpha s}{1-s\bar{n}}\cdot\sigma_n^2 + 2\eta_0 - (\nu-\eta)\bar{n} \\
\frac{d\sigma_n^2}{dt} &= -\frac{\alpha s}{1-s\bar{n}}\cdot\sigma_n^2\left(\frac{2\sigma_n^2-\bar{n}}{\bar{n}}\right) + 2\eta_0 + (\nu+\eta)\bar{n} - 2(\nu-\eta)\sigma_n^2\,.
\end{aligned}
$$

Setting the left sides to zero and clearing the denominators gives

$$
\begin{aligned}
0 &= -\alpha s\sigma_n^2 + \left[2\eta_0 - (\nu-\eta)\bar{n}\right]\left[1-s\bar{n}\right] \\
0 &= -\alpha s\sigma_n^2\left(2\sigma_n^2-\bar{n}\right) + \left[2\eta_0 + (\nu+\eta)\bar{n} - 2(\nu-\eta)\sigma_n^2\right]\bar{n}\left[1-s\bar{n}\right]\,.
\end{aligned}
$$

Assuming asymptotic expansions of the form $\bar{n} = \bar{n}_0 + s\bar{n}_1 + \cdots$ and $\sigma_n^2 = \sigma_0^2 + s\sigma_1^2 + \cdots$, we obtain

$$
\begin{aligned}
0 &= -\alpha s(\sigma_0^2 + s\sigma_1^2 + \cdots) + [2\eta_0 - (\nu-\eta)(\bar{n}_0 + s\bar{n}_1 + \cdots)][1 - s(\bar{n}_0 + s\bar{n}_1 + \cdots)] \\
0 &= -\alpha s(\sigma_0^2 + s\sigma_1^2 + \cdots)[2(\sigma_0^2 + s\sigma_1^2 + \cdots) - (\bar{n}_0 + s\bar{n}_1 + \cdots)] \\
&+ [2\eta_0 + (\nu+\eta)(\bar{n}_0 + s\bar{n}_1 + \cdots) - 2(\nu-\eta)(\sigma_0^2 + s\sigma_1^2 + \cdots)][\bar{n}_0 + s\bar{n}_1 + \cdots][1 - s(\bar{n}_0 + s\bar{n}_1 + \cdots)]\,.
\end{aligned}
$$

The zeroth order equations are

$$
\begin{aligned}
0 &= -\left[2\eta_0 - (\nu-\eta)\bar{n}_0\right] \\
0 &= \left[2\eta_0 + (\nu+\eta)\bar{n}_0 - 2(\nu-\eta)\sigma_0^2\right]\bar{n}_0\,.
\end{aligned}
$$

Assuming $\bar{n}_0 > 0$, we find

$$
\begin{aligned}
\bar{n}_0 &= 2\eta_0/(\nu-\eta) \\
\sigma_0^2 &= \left[2\eta_0 + (\nu+\eta)\bar{n}_0\right]/\left[2(\nu-\eta)\right] = 2\eta_0\nu/(\nu-\eta)^2\,,
\end{aligned}
$$

consistent with the neutral model, Eqs (27–28) of the main text. The first-order equations are

$$
\begin{aligned}
0 &= -\alpha\sigma_0^2 - (\nu-\eta)\bar{n}_1 - [2\eta_0 - (\nu-\eta)\bar{n}_0]\bar{n}_0 \\
0 &= -\alpha\sigma_0^2[2\sigma_0^2 - \bar{n}_0] + [(\nu+\eta)\bar{n}_1 - 2(\nu-\eta)\sigma_1^2]\bar{n}_0 + [2\eta_0 + (\nu+\eta)\bar{n}_0 - 2(\nu-\eta)\sigma_0^2][\bar{n}_1 - \bar{n}_0^2]\,.
\end{aligned}
$$

In the first equation, the expression in brackets evaluates to zero, so

$$
\bar{n}_1 = -\alpha\sigma_0^2/(\nu-\eta) = -2\alpha\eta_0\nu/(\nu-\eta)^3
$$

After some algebra, we find that the second equation yields,

$$
\sigma_1^2 = -\frac{2\alpha\nu\eta_0\,(\nu+\eta)}{(\nu-\eta)^4}\,.
$$

The above expressions may be combined to form the following two-term approximation for the mean and variance of TE load,

$$
\begin{aligned}
\bar{n} &\approx \frac{2\eta_0}{\nu-\eta} - \frac{2\alpha s\nu\eta_0}{(\nu-\eta)^3} = \frac{2\eta_0}{\nu-\eta}\left[1 - \alpha s\frac{\nu}{(\nu-\eta)^2}\right] \\
\sigma_n^2 &\approx \frac{2\nu\eta_0}{(\nu-\eta)^2} - \frac{2\alpha s\nu\eta_0\,(\nu+\eta)}{(\nu-\eta)^4} = \frac{2\nu\eta_0}{(\nu-\eta)^2}\left[1 - \alpha s\frac{(\nu+\eta)}{(\nu-\eta)^2}\right]\,.
\end{aligned}
$$

Because $v/(\nu-\eta)^2 > 0$, the equation for $\bar{n}$ indicates that weak selection decreases the mean TE load, consistent with our intuition. In the equation for σ_n^2, the factor $(\nu+\eta)/(\nu-\eta)^2$ is positive, so we conclude that weak selection also decreases the population variance. As for the index of dispersion, this analysis indicates that under weak selection the Fano factor is well-approximated by

$$\frac{\sigma_n^2}{\bar{n}} \approx \frac{\nu}{\nu-\eta} \cdot \frac{1-\alpha s(\nu+\eta)/(\nu-\eta)^2}{1-\alpha s\nu/(\nu-\eta)^2} = \frac{\nu}{\nu-\eta}\left[1-\alpha s\frac{\eta}{(\nu-\eta)^2}\right].$$

That is, under weak selection, the Fano factor is expected to decrease, because weak selection causes variance to decrease more than the mean.

5.3 Beta-binomial closure

For comparison to the negative binomial closure, we have worked through the possibility of choosing ψ to be the function that would be correct if the actual distribution of TE loads were beta-binomial distributed. The probability mass function for a beta-binomial random variable, $\mathbf{X} \sim \mathrm{BB}(\alpha,\beta)$ for $\alpha > 0$ and $\beta > 0$ on the interval 0 to m is

$$\mathsf{P}[\mathbf{X}=k] = \binom{m}{k}\frac{B(k+\alpha, m-k+\beta)}{B(\alpha,\beta)}, \quad k \in \{0,1,\ldots,m\}. \tag{A38}$$

where $B(a,b) = \int_0^1 t^{a-1}(1-t)^{b-1}\,dt$ is the beta function. The first three raw moments of a beta-binomial random variable are

$$\mu_1 = \frac{m\alpha}{\alpha+\beta} \tag{A39}$$

$$\mu_2 = \frac{m\alpha[m(1+\alpha)+\beta]}{(\alpha+\beta)(1+\alpha+\beta)} \tag{A40}$$

$$\mu_3 = \frac{m\alpha[m^2(1+\alpha)(2+\alpha)+3m(1+\alpha)\beta+\beta(\beta-\alpha)]}{(\alpha+\beta)(1+\alpha+\beta)(2+\alpha+\beta)}, \tag{A41}$$

while the variance is

$$\hat{\mu}_2 = \mu_2 - \mu_1^2 = \frac{m\alpha\beta(\alpha+\beta+m)}{(\alpha+\beta)^2(\alpha+\beta+1)}.$$

Using Eq (A22) it can be shown that the third central moment of a beta-binomial random variable is

$$\hat{\mu}_3 = \hat{\mu}_2\frac{(\alpha+\beta+2m)(\beta-\alpha)}{(\alpha+\beta)(\alpha+\beta+2)}.$$

Inverting Eqs (A39–A40) gives

$$\alpha = \frac{m\mu_1-\mu_2}{m(\mu_2/\mu_1-\mu_1-1)+\mu_1}$$

$$\beta = \frac{(m-\mu_1)(m-\mu_2/\mu_1)}{m(\mu_2/\mu_1-\mu_1-1)+\mu_1}.$$

From these values we calculate the following algebraic relationship for the third central moment in terms of the mean and variance of the haploid TE load,

$$\hat{\mu}_3 = \hat{\mu}_2\frac{(m-2\mu_1)(\mu_1^2-m\mu_1-\hat{\mu}_2+2m\hat{\mu}_2)}{m\mu_1(m-\mu_1-2)+\hat{\mu}_2 m+2\mu_1^2} =: \psi_{BB}(\hat{\mu}_2,\mu_1). \tag{A42}$$

For the mean and variance of the diploid TE load, the corresponding expression is

$$\mathsf{E}[(\mathbf{n}-\bar{n})^3] = 2\hat{\mu}_3 = \sigma^2 \frac{(m-\bar{n})(\bar{n}^2 - 2m\bar{n} - 2\sigma^2 + 4m\sigma^2)}{m\bar{n}(2m-\bar{n}-4) + 2m\sigma^2 + 2\bar{n}^2} \,. \tag{A43}$$

This beta-binomial closure represented by this expression for the third central moment of diploid TE load is arguably preferable to the negative binomial closure discussed in Sec 5.1, because a beta-binomial random variable has finite support, that is, it values between 0 and m as in Eq (A38). On the other hand, the negative binomial closure results in a simpler expression that often gives approximately the same nullclines and solution trajectories, as in Fig 7 of the main text. It is notable that the expression for $\mathsf{E}[(\mathbf{n}-\bar{n})^3]$ obtained using the beta-binomial distribution, Eq (A43), is well-approximated by the negative binomial result, Eq (A37), when the number of loci are not limiting ($\bar{n} << m$). To see this, one may compare Eq (A36) with Eq (A42) and show that $\psi_{BB}(\hat{\mu}_2, \mu_1) \to \psi_{NB}(\hat{\mu}_2, \mu_1)$ as $m \to \infty$.

6 Numerical methods

The master equation model given by Eq (31) of the main text is a system of $m+1$ ordinary differential equations. When m is sufficiently small, it is straightforward to use a relaxation method to calculate the limiting probability distributions for the master equation. Because the number of ODEs in the master equation grows with the number of occupiable loci, it can be more efficient, especially when m is large, to numerically solve for the limiting probability distribution of the associated Fokker-Planck equation. Writing $\rho(n,t)\,dn = \Pr[n \le \mathbf{n} \le n + dn]$ for the time-dependent probability density function for TE load, $\rho(n,t)$ solves the following Fokker-Planck equation [2, 3],

$$\frac{\partial \rho}{\partial t} = -\frac{\partial}{\partial n}\left[a(n)\rho\right] + \frac{1}{2}\frac{\partial^2}{\partial n^2}\left[b(n)\rho\right] \,. \tag{A44}$$

In this expression, $\mathbf{n}$ is the random variable (the TE load of a randomly sampled haploid genome) and n is the independent variable of the probability density $\rho(n,t)$. The drift and diffusion terms of the Fokker-Planck equation are

$$a(n) \;=\; -v(n) + u(n) = \eta_0 - \left(\nu - \eta + \frac{\eta_0}{m}\right) n - \frac{\eta}{m} n^2 \tag{A45}$$

$$b(n) \;=\; v(n) + u(n) = \eta_0 + \left(\nu + \eta - \frac{\eta_0}{m}\right) n - \frac{\eta}{m} n^2 \,, \tag{A46}$$

where we have used the gain and loss terms $u(n) = (\eta_0 + \eta n)(1 - n/m)$ and $v(n) = \nu n$ as in Eqs (18–19) of the main text. Writing Eq (A44) in conservative form as $\partial\rho/\partial t = -\partial\phi/\partial n$, where $\phi(n)$ is the probability flux, we find

$$\phi(n) = a(n)\rho - \frac{1}{2}\frac{\partial}{\partial n}[b(n)\rho] \,.$$

For a steady-state solution $\hat{\rho}(n)$ with no-flux (Neumann) boundary conditions, setting $\phi(n) = 0$ leads to the analytical solution

$$\hat{\rho}(n) = \frac{\theta}{b}\exp\left(2U\right) \quad \text{where} \quad U(n) = \int_0^n \frac{a(n')}{b(n')}\,dn' \,, \tag{A47}$$

$a(n)$ and $b(n)$ are given by Eqs (A45–A46), and θ is a normalization constant such that $\int \hat{\rho}(n)\, dn = 1$. In fact, $U(n)$ may be any antiderivative satisfying $U' = a/b$, because the normalization constant θ absorbs the arbitrary constant of integration.

Several numerical methods were used to simulate the models of TE population dynamics defined by the master equation for the neutral model, Eqs (20–22) of the main text, and the master equation that accounts for selection, Eq (31). Fig 4 of the main text used a flux-limiting numerical scheme and the method of lines to integrate the Fokker-Planck equation, Eq (A44), until a limiting value was reached. Fig 5 was obtained using the analytical steady state of the Fokker-Planck equation given by Eq (A47). Fig 6 used Monte Carlo simulation of drift-diffusion process associated to the Fokker-Planck equation, Eq (A44). Comparing these results to the flux-limiting numerical scheme revealed that, for some parameter sets that included strong selection, the infinite population model exhibits periodic solutions that are rarely observed in large finite populations. Fig 7 was calculated using the moment equations with selection and negative binomial moment closure, Eqs (41–42) (main text). Beta-binomial moment closure leads to very similar results unless m is quite small (on the order of 100). Fig 8 of the main text used moment equations with selection and beta-binomial closure, i.e., Eqs (33–34) with Eq (39) (main text).

References

1. Charlesworth B, Charlesworth D. The population dynamics of transposable elements. Genetics Research. 1983;42(1):1–27.

2. Gardiner C. Stochastic Methods: A Handbook for the Natural and Social Sciences. 4th ed. Springer; 2009.

3. Van Kampen NG. Stochastic Processes in Physics and Chemistry. 3rd ed. North Holland; 2007.
